# Development of somites and their derivatives in amphioxus, and implications for the evolution of vertebrate somites

**DOI:** 10.1186/s13227-015-0007-5

**Published:** 2015-05-14

**Authors:** Jennifer H Mansfield, Edward Haller, Nicholas D Holland, Ava E Brent

**Affiliations:** Department of Biology, Barnard College, Columbia University, 3009 Broadway, New York, NY 10027 USA; Department of Integrative Biology, University of South Florida, 4202 East Fowler Avenue, Tampa, FL 33620 USA; Marine Biology Research Division, Scripps Institution of Oceanography, University of California at San Diego, 9500 Gilman Drive, La Jolla, CA 92093 USA

**Keywords:** Somite evolution, Sclerotome, Skeleton, Connective tissue, Tendon, Amphioxus, Chordate

## Abstract

**Background:**

Vertebrate somites are subdivided into lineage compartments, each with distinct cell fates and evolutionary histories. Insights into somite evolution can come from studying amphioxus, the best extant approximation of the chordate ancestor. Amphioxus somites have myotome and non-myotome compartments, but development and fates of the latter are incompletely described. Further, while epithelial to mesenchymal transition (EMT) is important for most vertebrate somitic lineages, amphioxus somites generally have been thought to remain entirely epithelial. Here, we examined amphioxus somites and derivatives, as well as extracellular matrix of the axial support system, in a series of developmental stages by transmission electron microscopy (TEM) and *in situ* hybridization for collagen expression.

**Results:**

The amphioxus somite differentiates medially into myotome, laterally into the external cell layer (a sub-dermal mesothelium), ventrally into a bud that forms mesothelia of the perivisceral coelom, and ventro-medially into the sclerotome. The sclerotome forms initially as a monolayered cell sheet that migrates between the myotome and the notochord and neural tube; subsequently, this cell sheet becomes double layered and encloses the sclerocoel. Other late developments include formation of the fin box mesothelia from lateral somites and the advent of isolated fibroblasts, likely somite derived, along the myosepta. Throughout development, all cells originating from the non-myotome regions of somites strongly express a fibrillar collagen gene, *ColA,* and thus likely contribute to extracellular matrix of the dermal and axial connective tissue system.

**Conclusions:**

We provide a revised model for the development of amphioxus sclerotome and fin boxes and confirm previous reports of development of the myotome and lateral somite. In addition, while somite derivatives remain almost entirely epithelial, limited de-epithelialization likely converts some somitic cells into fibroblasts of the myosepta and dermis. Ultrastructure and collagen expression suggest that all non-myotome somite derivatives contribute to extracellular matrix of the dermal and axial support systems. Although amphioxus sclerotome lacks vertebrate-like EMT, it resembles that of vertebrates in position, movement to surround midline structures and into myosepta, and contribution to extracellular matrix of the axial support system. Thus, many aspects of the sclerotome developmental program evolved prior to the origin of the vertebrate mineralized skeleton.

## Background

In vertebrates, the somites give rise to musculoskeletal tissues, including the bones and cartilage of the vertebral column and its associated muscles and connective tissue. Somites form via mesenchymal-to-epithelial transition of the presomitic mesoderm. Subsequently, epithelial somites undergo EMT and become subdivided into compartments with distinct tissue fates (reviewed in [1,2]). The dermomyotome gives rise to myotome, which contains skeletal muscle progenitors, and to central dermomyotome, which in turn gives rise to the dorsal dermis. Sclerotome contains the progenitors for cartilage and bone of the vertebral column. The sclerotome further subdivides to form syndetome, which gives rise to axial tendons. Somite compartmentalization and tissue fates are largely shared across vertebrates. However, comparison across groups of higher vertebrates reveals substantial variation in the position, relative sizes, and inductive mechanisms of the somitic compartments [1,3-6]. These differences in turn contribute to variation in the position and size of musculoskeletal tissues, which allows functional specializations among different vertebrate groups [7].

Somites evolved prior to the origin of vertebrates and can be traced back at least to ancestral invertebrate chordates [8,9]. However, the extent to which ancestral somites were compartmentalized, and the evolutionary history of their organization, is unclear. Further, within the chordates, mineralized skeletal tissue is an evolutionary novelty of vertebrates, raising the question of how a compartment of skeletal precursor cells arose. Comparison of vertebrate somites to those of extant, basal taxa can help to address these questions. Here, we examine somite development in a chordate that diverged from the ancestors of vertebrates prior to the origin of skeletal tissue: the cephalochordate amphioxus. The other group of invertebrate chordates, the tunicates, cannot be used in such a comparison, because they have secondarily lost segmentation [10].

**Figure 1:**
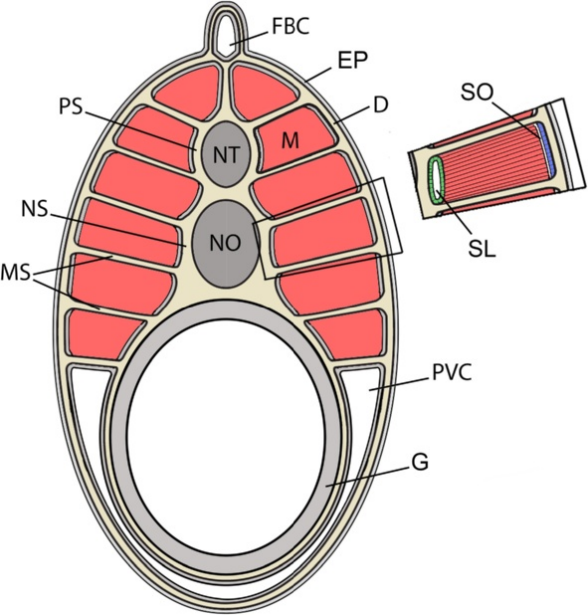
Morphology of muscle segments in adult amphioxus. Schematic transverse section shows the muscle (M) segments (myomeres) in red and the axial and dermal extracellular connective tissue in tan. Because adult segments are chevron shaped, multiple myosepta (MS), which are present at their anterior and posterior borders of each segment, are observed in a transverse section. Mesothelial cells (grey) surround the muscle segments and enclose the fin box and perivisceral coeloms. Inset details the structure of the mesothelia surrounding muscle segments; the medial mesothelium (green) is double layered and encloses the sclerocoel (SL). The lateral mesothelium (blue) is single layered and separated from the muscle by the somitocoel (SO). Abbreviations: D, dermis; FBC, fin box coelom; M, muscle; MS, myoseptum; NO, notochord; NS, notochordal sheath; NT, neural tube; PS, perineural sheath; PVC, perivisceral coelom; SL, sclerocoel; SO, somitocoel; EP, epidermis.

**Figure 2:**
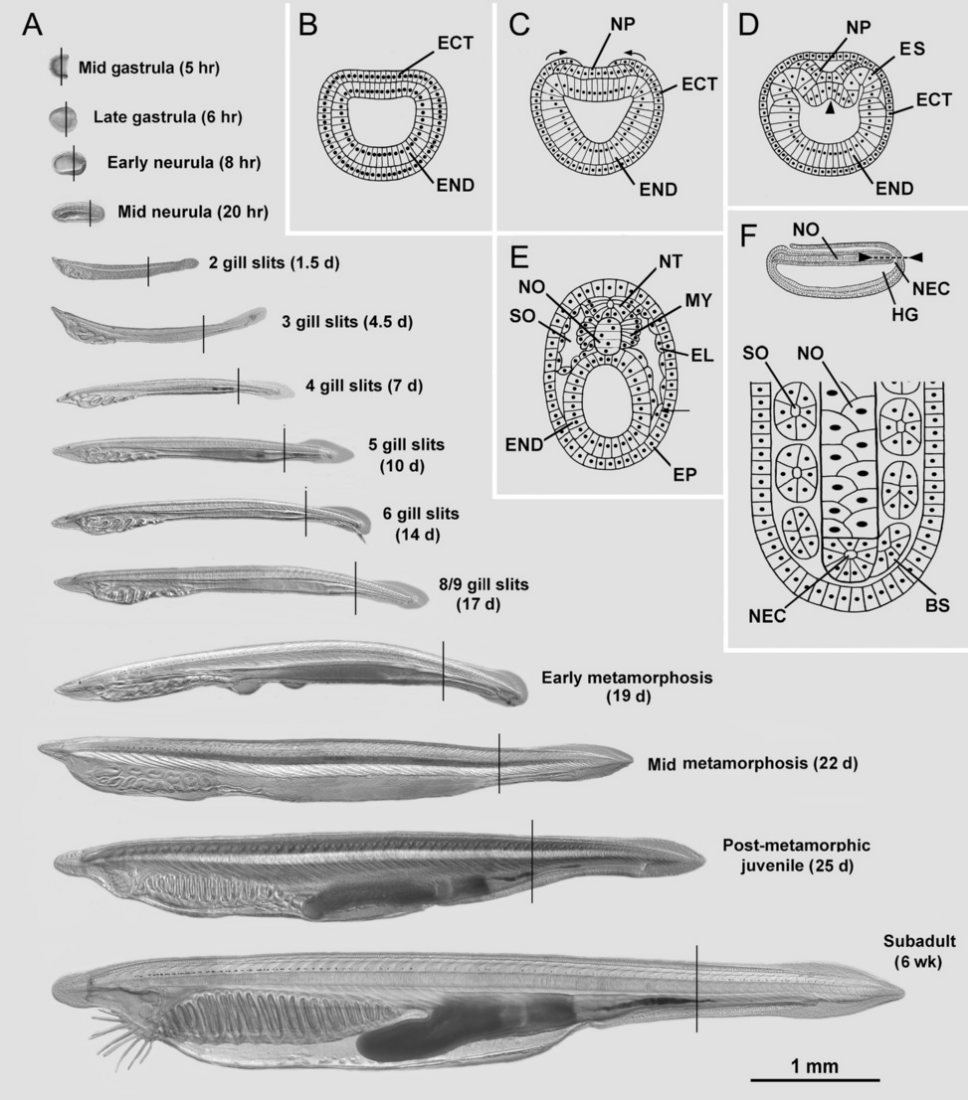
Overview of amphioxus development and the stages examined in this study. **(A)** Differential interference contrast micrographs of living specimens in side view with anterior to the left (with times after fertilization); the present study was limited to studying the body region indicated by the vertical line on each specimen; the 1-mm scale line applies to all images. **(B-E)** Schematic drawings of transverse sections of embryos and early larvae. **(B)** Late gastrula. **(C)** Gastrula-to-neurula transition (arrows indicate epidermis overgrowing neural plate during the first phase of neurulation). **(D)** Early neurula (arrowhead indicates initial stage of evagination of the notochord mid-dorsally from the endoderm). **(E)** Mid neurula (arrow indicates the ventral somitocoel extension that later pinches off to give rise to the perivisceral coelom). **(F)** Formation of more posterior somites occurs through segmentation from the neurenteric canal. Drawings B-E are reproduced from Hatschek [14,27], respectively. Abbreviations: BS, budding somite; ECT, ectoderm; EL, external cell layer; END, endoderm; EP, epidermis; ES, epithelial somite; HG, hindgut; MY, myotome; NEC, neurenteric canal; NO, notochord; NP, neural plate; NT, neural tube.

Amphioxus and vertebrates have several features in common, including pharyngeal slits, a dorsal nerve cord and notochord, and segmented axial musculature derived from somites [11,12]. In adult amphioxus (Figure 1), muscle segments (myomeres) extend along the length of the body axis. Although amphioxus lacks skeletal tissue, collagen-based connective tissues with few or no cells are found in the positions where the vertebrate axial skeleton forms. The myomeres are separated by collagenous myosepta that are continuous laterally with the sub-epidermal collagen layer (dermis) and medially to the collagenous layers ensheathing the axially located notochord and nerve cord: the notochordal and perineural sheaths. As myomeres contract sequentially during the undulatory movement of swimming, the generated force is transmitted to the notochord via these axial connective tissues [7]. Cephalochordates, then, most closely represent both the ancestral segmented body plan of vertebrates, and the anatomical features of the most basal somites. The morphology of the adult musculoskeletal system has been extensively studied in amphioxus. However, previous studies of connective tissues and/or the fates of somite derivatives have focused on limited developmental stages [12-18] or the adult stage alone [19-23], and because of this limited sampling, there is as yet no connected account of their development. Therefore, in the present study, we frequently sampled a developing culture of amphioxus, ranging from early embryos through subadults (Figure 2A).

The early stages of amphioxus somite formation are somewhat different from vertebrates. The most anterior somites are produced by evagination from the gut wall (Figure 2B,C,D); those added later form directly from the epithelium surrounding the neurenteric canal (Figure 2F) without the interposition of presomitic mesoderm found in vertebrates [24,25]. The terminology for somite-derived cell lineages within the chordates has not been well standardized, so the terms used here are presented in Table 1. Both Kowalevski [26] and Hatschek [27] accurately described the initial division of amphioxus somites into myotome (medial) and non-myotome (lateral) compartments (Figure 2E), but the subsequent behaviors of cells, particularly the latter population, are poorly understood. Although the non-myotome compartment(s) do not give rise to skeleton, Hatschek proposed that they give rise to the axial connective tissue system and consist of two populations. The first, located laterally, differentiates in place into the lateral mesothelial layer that produces the dermis; this has been referred to the dermatome, although its correspondence with vertebrate dermatome has not been clearly established. Hatschek proposed that the second population, located ventro-medially, is homologous to vertebrate sclerotome, evaginates as a hollow diverticulum that migrates toward the midline, and gives rise to the notochordal and perineural sheaths. Hatschek’s version of somite development has been largely accepted [3,12,13,15,28], but sometimes challenged [17,18]; however, it has not been revisited by examining a series of developmental stages, or with molecular data. Further, the ultimate fates amphioxus non-myotome cells have not been shown. Although myotome derivatives have been followed with a molecular marker [29], no such marker has yet been used to track the non-myotome cells. In addition, it has been proposed that the amphioxus perivisceral coelom and fin box mesothelia arise as epithelial evaginations from the non-myotome compartment of the somite, but this has not yet been clearly demonstrated [30,31].

**Table 1:** **Synonyms for somite-related structures of amphioxus, anamniotes, and amniotes**

**Group**	**Structure**						
Amphioxus	*Somitocoel* ^1^		*Myotome* ^4^	*External cell layer* ^1^	*Sclerotome* ^1^	*Sclerocoel* ^9^	*Lateral plate 10*
	Myocoel^2^	No equivalent	Myomesothelium^5^	Lateral wall^6^	Sclerablatt^3^		Seitenplatt^3^
	Myocöl^3^		Muskelblatt^3^	Dermomyotome^7^			
				Dermatome^8^			
				Cutisblatt^3^			
Anamniote	*Somitocoel* ^11^	Dermomyotome	*Myotome* ^13^	*External cell layer* ^11,14^	*Sclerotome* ^18^	No equivalent	*Lateral plate 19*
	Myocoel^12^		Primary myotome^11^	Dermomyotome^15^			
				Dermatome^16^			
				Dermoendothelium^17^			
Amniote	*Somitocoel* ^20^	*Dermomyotome* ^21^	*Myotome* ^21^	*Central dermomyotome* ^21^	*Sclerotome* ^21^	No equivalent	*Lateral plate 21*
				Dermatome^22^			

Terms used in the present paper are *in italics*. ^1^Present paper (without precedent). ^2^ [15,68]. ^3^ [14]. ^4^ [3]; later in development, the myotome is generally termed ‘trunk muscle’. ^5^ [15]. ^6^ [15,68]. ^7^ [3]. ^8^ [69]. ^9^ [15]; ([14], considered this space as a subdivision of the myocoel). ^10^ [30]. ^11^ [70]. ^12^ [3]. ^13^ [71]; [3] later in development, the myotome is generally termed ‘trunk muscle’. ^14^[3] use the term ‘external cell layer’ alternatively with ‘lateral wall cells’. ^15^ [5]. ^16^ [72]. ^17^ [73] (name indicating later development as scale pockets in the dermis). ^18^ [71]; ([3] a region of sclerotome-derived cells (collectively the syndetome) gives rise to the tenocytes (myoseptal cells) at myotendinous junctions [5,38]). ^19^ [74]. ^20^We will consider all chordate somitocoels homologous, although [3] have questioned that homology. The loose cells (collectively the arthrotome) in the amniote somitocoel are evidently sclerotome-related and give rise to the articular surfaces on the amniote axial skeleton [3]. ^21^ [33] Later in development, the myotome is generally termed ‘trunk muscle’. ^22^ [75].

Here, we examine the development of amphioxus somites in a fine-grained sampling of developmental timepoints. Our aims are to provide a description that can inform models of somite and sclerotome evolution, as well as a foundation upon which future molecular studies of amphioxus somite compartmentalization can build. First, we examined somites and their derivatives in closely spaced samples with transmission electron microscopy (TEM) during the embryonic, larval, and subadult stages. We also examined development of extracellular connective tissue layers, all of which we show form in close apposition to cells derived from non-myotome somite lineages. Finally, we provide evidence that non-myotome somite cells are secretory, connective tissue-producing cells, based on ultrastructure and their expression of the single clade A fibrillar collagen gene of amphioxus (*ColA*) at all stages of development. In vertebrates, clade A collagen gene family members are expressed in somite-derived connective tissues including the dermis, cartilage, bone, and tendon [32-34]. We propose a revised scheme for amphioxus somite morphogenesis and cell fate and, in the light of these data, compare somite derivatives between amphioxus and vertebrates and consider their possible homologies.

## Methods

### Amphioxus collection and culture

Amphioxus adults were collected from Tampa Bay during the summers of 2010 and 2012 using a shovel and sieve. Adults were induced to spawn in the lab, and embryos and larvae were cultured at 20°C as previously described [35]. Developmental stages were fixed for TEM or *in situ* hybridization at a dozen time intervals covering the period from the gastrula through the subadult. Such a comprehensive study at the TEM level is a major undertaking, and to keep it within bounds, we limited our coverage to a body region about three-fourths of the way between the anterior and posterior ends of the body (depicted as vertical lines on each animal, Figure 1A). A section at this level avoids the structural complexity of the atrial region as it develops.

### TEM

For each developmental stage sampled, half a dozen animals were fixed in 3% glutaraldehyde in 0.1% phosphate buffer (pH 7.3) with 0.45 M sucrose for 2 h at room temperature. Specimens were rinsed in three 5-min changes of 0.1 M phosphate buffer (pH 7.3) with 0.45 M sucrose and then postfixed in 1% osmium tetroxide at 3°C for 1 h. The specimens were then dehydrated in an ethanol series, transferred to propylene oxide, and embedded in LX-112 resin. For orientation, 0.5-μm-thick sections were cut and stained with 1% toluidine blue. For thin sectioning, contrast of gold sections was enhanced with uranyl acetate and lead citrate. The following numbers of specimens were observed at each stage: mid gastrula (1), late gastrula (1), early neurula (1), mid-late neurula (3), 2 GS larva (3), 3 GS larva (2), 4 GS larva (1), 5 GS larva (2), 6 GS larva (1), 7/8 GS larva (1), 9 GS larva (1), early metamorphic (3), postmetamorphic juvenile (6), subadult (7).

### mRNA *in situ* hybridization

For embryos and larvae, whole-mount *in situ* hybridization was performed as described previously [36]. After probe detection, embryos were incubated in 1 μg/mL DAPI (Sigma, St. Louis, MO, USA) for 10 min and washed in PBT. Embryos were embedded in gelatin and frozen as described in [37] and 3-μm sections cut on a Leica cryostat (Leica Microsystems, Wetzlar, Germany). Larvae were dehydrated through a graded series from PBS to ethanol, equilibrated in 50/50 ethanol/Spurr’s resin in a rocking desiccation chamber, washed 4 × 30 min in Spurr’s resin under desiccation, aligned in rubber molds, and polymerized at 68°C overnight. Spurr’s resin (Sigma EM0300; Sigma, St. Louis, MO, USA) was prepared according to manufacturer’s instructions with the following proportions of reagents: 4.1 g ERL, 1.75 g DER, 5.9 g NSA, 0.1 g DMAE. Sections (3 μm) were cut with a glass knife on a (model) microtome or with a tungsten-carbide knife on a rotary microtome (Leica RM225; Leica Microsystems, Wetzlar, Germany). For adults, tissues were embedded in paraffin and sectioned into 10-μm sections, and section *in situ* hybridization was performed, all as described in [38]. *ColA* and *MLC* probes were previously described [29,36]. Specimens were photographed under oil on a Nikon Axiophot microscope with a Nikon DigiSight camera (Nikon, Tokyo, Japan).

## Results

### Morphology and fate of the somitic compartments

**Figure 3:**
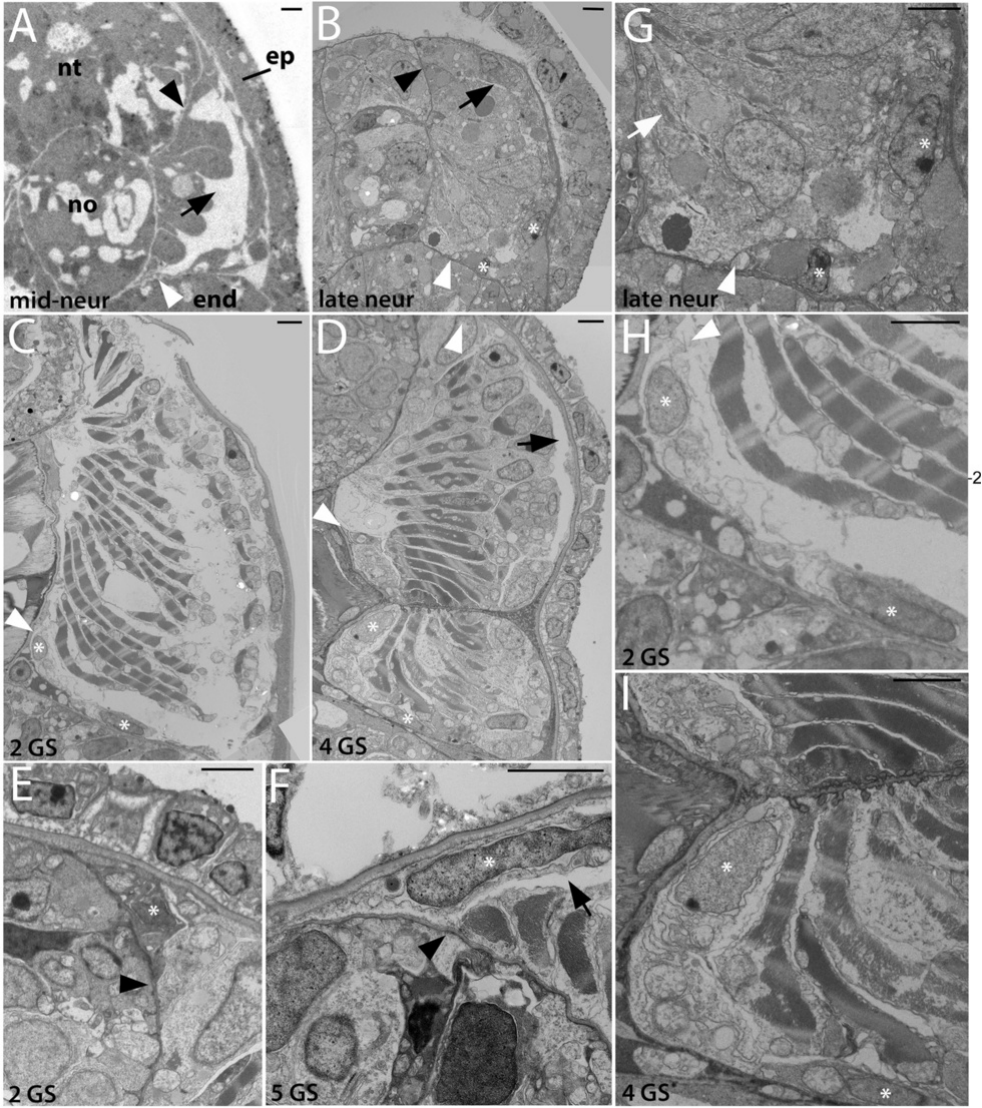
Development of the non-myotome somite (1): embryos and early larval stages. Transmission electron micrographs show the position of the non-myotome cells (external cell layer and sclerotome) relative to myotome at the stages indicated. **(A-D)** are whole somite views showing the progressive change in the position of the sclerotome. In these and all panels, white arrowheads mark the border between sclerotome and the myotome and white asterisks mark sclerotome or external cell layer nuclei. Black arrowheads mark the transition between dorsal myotome and the external cell layer and black arrows indicate the somitocoel **(G-I)** are details of panels (B-D), respectively, focusing in the position of the sclerotome-myotome boundary. White arrow in (G) indicates muscle fibers. **(E, F)** are details of the dorsal myotome-external cell layer boundary. Scale bars are 2 μm, dorsal is up, medial is to the left. Structures are labeled on the first panel only: end, endoderm; epi, epidermis; no, notochord; nt, neural tube. Stage abbreviations: neur, neurula; GS, gill slit.

**Figure 4:**
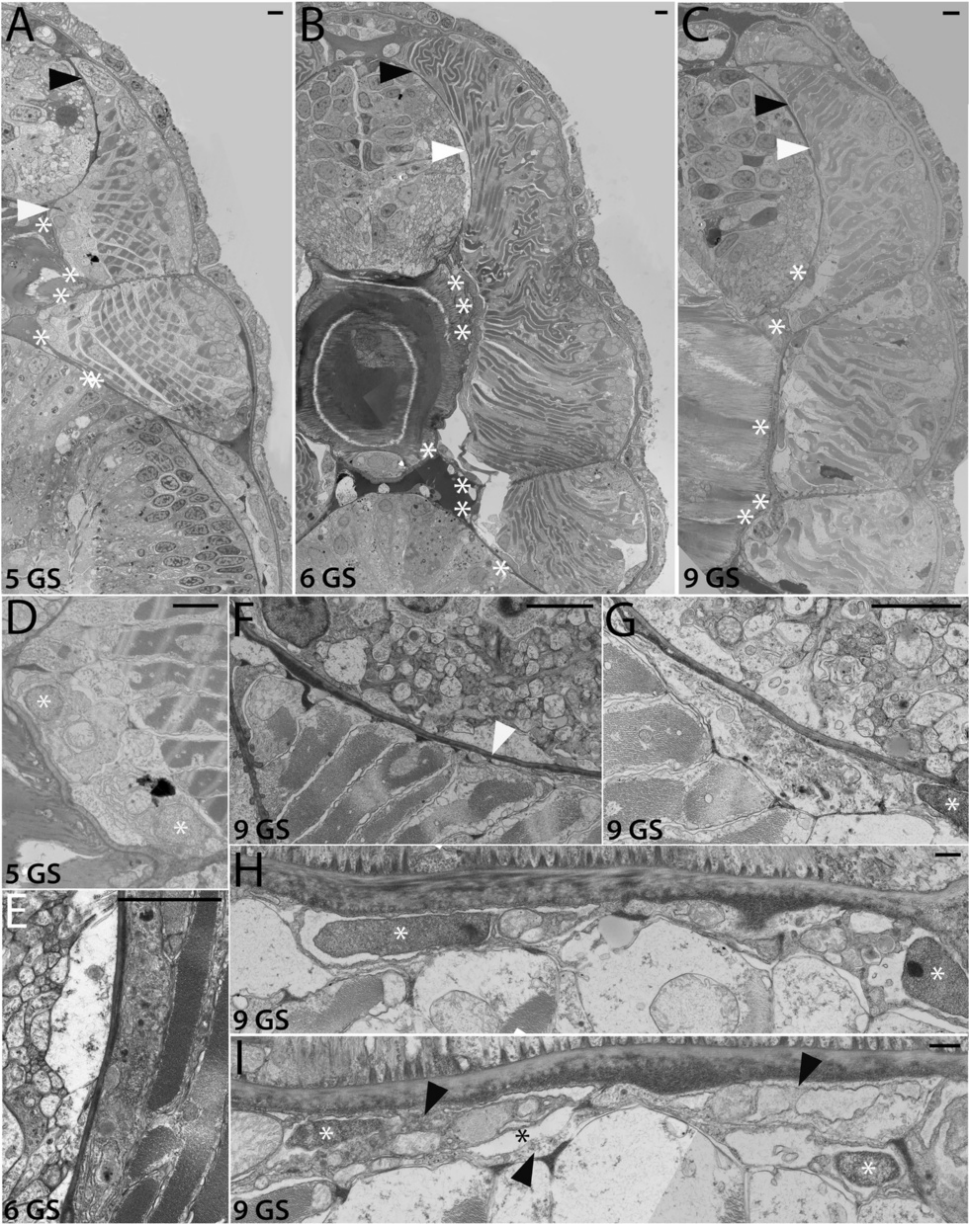
Development of the non-myotome somite (2): mid to late larval stages. Transmission electron micrographs show the position of the myotome and the non-myotome (external cell layer and sclerotome) cells at the stages indicated. **(A-C)** are whole somite views that show the progressive change in position of the sclerotome. In these and panels, white arrowheads mark the border between sclerotome and myotome, and black arrowheads mark the border between the dorsal myotome and external cell layer. White asterisks show positions of sclerotome nuclei beside the midline structures. **(D)** is a detail of **(A)** showing the sclerotome-myotome boundary. (**E)** is a detail of **(B)** showing sclerotome beside the neural tube, **(F-H)** are details of **(C)** showing **(F)** the process of an external cell layer cell extended ventrally along the neural tube (arrowhead), **(G)** the nucleus of a sclerotome cell beside the notochord **(H)** a single-layered sclerotome along the notochord. (**I)** shows a different 9 GS specimen in which the sclerotome beside the notochord is double layered; black arrowheads in opposite directions indicate the two layers, black asterisk marks the sclerocoel. **(A-E)**, dorsal is up, medial is to the left. **(F-I)** are rotated 90° counterclockwise, with dorsal to the left and medial up. Scale bars are 2 μm. Stage abbreviations: GS, gill slit.

**Figure 5:**
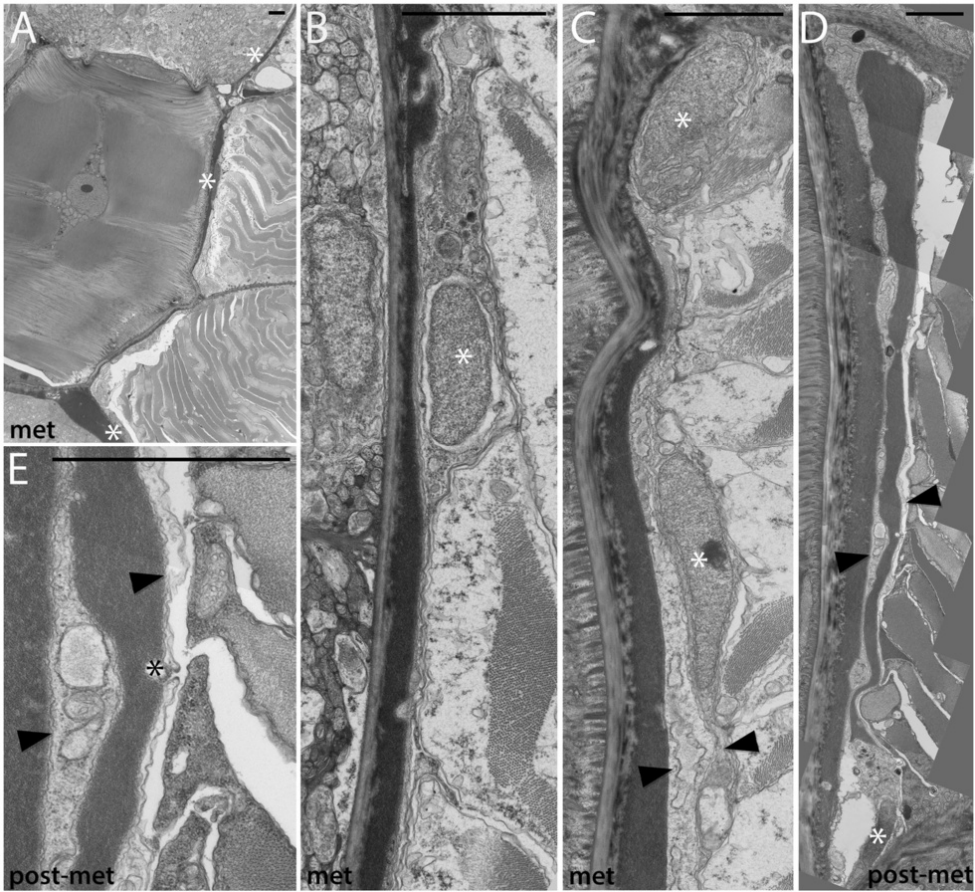
Development of the non-myotome somite (3): metamorphosis through adult stages. **(A)** Lower power view, showing sclerotome-derived mesothelium beside the notochord and a portion of the neural tube. Sclerotome nuclei are marked by white asterisks in all panels. **(B, C)** Higher power views of the sclerotome-derived mesothelium beside the neural tube **(B)** and notochord **(C). (D)** The double-layered sclerotome beside the notochord is more easily visualized metamorphosis when it is often filled with fluid. Black arrowheads indicate the double layer at the notochord level in **(C)** and **(D). (E)** Detail of **(D)** showing an adherens junction between adjacent sclerotome cells (black asterisk). In all panels, dorsal is up, medial to the left; scale bars are 2 μm. Stage abbreviations: met, early metamorphosis, post-met, post-metamorphic juvenile.

**Figure 6:**
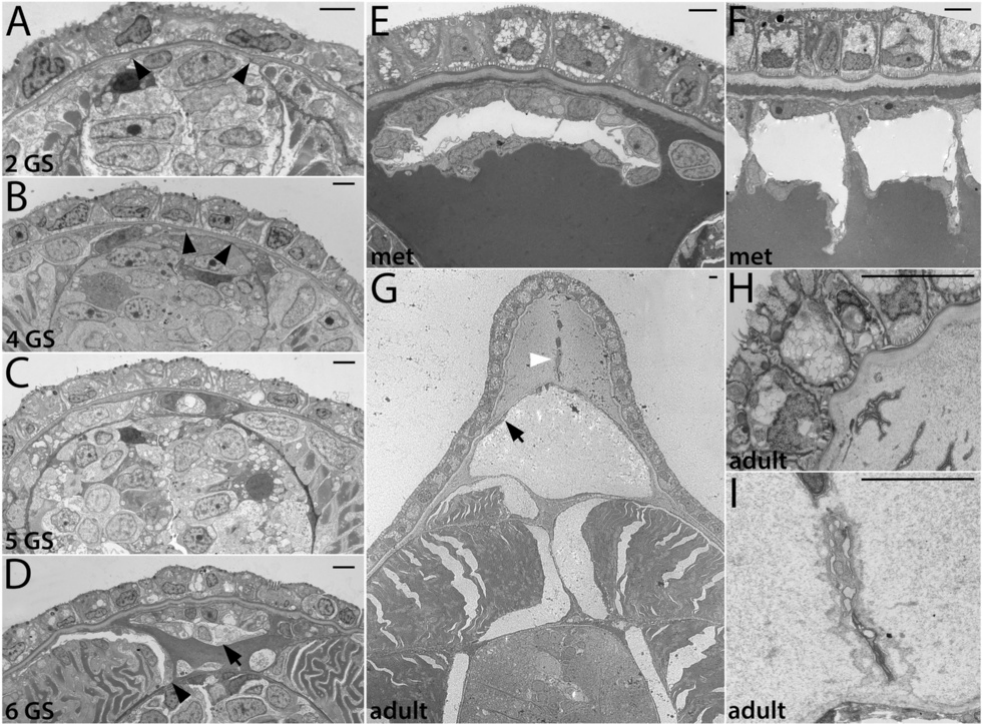
Fin box morphogenesis. The fin box mesothelium develops from the dorsal-most cells of the external cell layer. **(A, B)** Dorsal cells from a pair of somites extend over the neural tube (black arrowheads mark the boundary of external cell layer cells). **(C)** By mid larval stages, a continuous epithelium is present overlying the neural tube. (**D)** A double-layered fin box mesothelium (arrowhead) is apparent after it separates from the external cell layer (arrowhead marks the dorsal boundary between the external cell layer and myotome). **(E, F)** Expanded fin box coelom in late larval and metamorphic stages; the mesothelium completely encloses the coelom as shown in transverse **(E)** and sagittal **(F)** views. **(G)** The adult fin box mesothelium is single layered (arrow), and extracellular matrix also accumulates within the dorsal-most dermis separating the coelom and epidermis. White arrowhead indicates a cutaneous canal, which is also shown in detail in (**H, I).** Scale bars are 2 μm. Stage abbreviations: GS, gill slit; met, early metamorphosis, adult, subadult.

In this section, we examine the development and positions of the non-myotome lineages throughout development, shown in Figures 3, 4, 5, and 6. Some panels in these figures provide overviews of whole somites, while others show details specific to one somite region or derivative. In the text below, we focus on one somite-derived structure at a time and provide a connected account of its development.

#### A. Development of the sclerotome and the sclerocoel

We first examined development and fates of the ventral part of the somite, which we will hereafter refer to as sclerotome (Table 1; Figures 3, 4, and 5). Somites of mid-neurulae are epithelial spheres. The medial (myotome) cells have already begun to elongate along the medial-lateral axis (Figure 3A) and express muscle markers [29]. In contrast, the non-myotome, which includes ventral (sclerotome) and the lateral (external cell layer) regions, is a continuous, thin epithelium that borders the epidermal ectoderm and dorsal gut tube, respectively. The morphological difference between myotome and non-myotome is already pronounced at this stage (Figure 3A; arrowheads mark the transitions dorsally (black) and ventrally (white); somitocoel is indicated by a black arrow). In late neurulae (Figure 3B,G), myotome cells are further elongated, contain myofibers (white arrow), and largely fill the somitocoel (black arrow) in the preparation shown. In contrast, the non-myotome is a squamous epithelium that becomes further thinned during late embryonic and larval stages. The border between the ventral myotome and sclerotome (white arrowhead) is located along the gut, near but not abutting the notochord; asterisks in Figure 3B,G mark the nuclei of the two ventral-most sclerotome cells. During larval stages, the position of the sclerotome cells becomes progressively more medial, and then dorsal. In Figures 3 and 4, white arrowheads mark the sclerotome-myotome boundary. In 2 gill slit (GS) larvae, sclerotome cells extend medially to the notochord (Figure 3C,H), and at the 4 GS stage, they extend along the ventral length of the somite and partway up the notochord (Figure 3D,I). Note that because segments become chevron shaped, myosepta separating adjacent segments are apparent in transverse sections at this stage (Figure 3D). In 5 GS larvae, the sclerotome extends to the dorsal edge of the notochord (Figure 4A), and at the 6 GS stage, these cells extended processes (and in some specimens, nuclei) dorsally along the neural tube as well (Figure 4B). At the 9 GS stage, the sclerotome epithelium extends most of the way up the neural tube (Figure 4C, white arrowhead). By early metamorphic stages, and persisting in adults (Figure 5, Figure 6G, and see below), this epithelium derived from sclerotome becomes continuous with that derived from lateral sclerotome, such that a layer of mesothelium derived from non-myotome somites completely surrounds each muscle segment. Together, this suggests movement of the sclerotome medially toward and then dorsally along the midline structures, which would be similar to migration path of (mesenchymal) sclerotome in vertebrates. However, whether this occurs through active migration of sclerotome or the differential growth or migration of the myotome or other surrounding tissues cannot be determined from static images.

Hatschek [14,27] described the sclerotome as a double-layered diverticulum. However, our TEM series showed that from embryonic through mid larval stages, the sclerotome cells always comprise a single layer. Sclerotome cells in early through mid larval stages are shown beside the notochord in Figures 3H,I and 4D (sclerotome nuclei are marked by white asterisks). However, in late larvae and metamorophic animals, a double-layered epithelium is apparent at the notochord level (Figures 4I and 5C). In these images, the two layers are indicated by black arrowheads, sclerotome nuclei are marked by white asterisks, and the enclosed coelom, the sclerocoel, is marked by a black asterisk. However, in some sections, it appears single layered at the notochord level (Figure 4H). Later, in post-metamorphic animals, the sclerotome is invariably double layered and granular extracellular material is observed inside the sclerocoel (Figure 5E,D). We observed adherens junctions between these cells throughout development (for example, Figure 5E, black asterisk), as was previously described in adults [15], indicating that these sclerotome cells remain epithelial throughout development. Finally, while the sclerotome mesothelium becomes double layered at the notochord level, it apparently remains single layered at the level of the neural tube, at least through the latest stage we examined, the subadult. Sclerotome cells beside the neural tube are shown in Figures 4E,F,G and 5B.

We conclude that the ventral somite, or sclerotome, cells give rise to the mesothelium that encloses the sclerocoel and that separates the myotomes from midline structures, notochord and neural tube. This mesothelium is apposed to the extracellular collagen of the notochordal and perineural sheaths (see below). The morphogenesis of the sclerotome-derived mesothelium is somewhat different than has been previously been proposed (see ‘Discussion’).

#### B. Development of the external cell layer and somitocoel

In contrast to the progressive change in position of sclerotome cells, non-myotome cells from the lateral part of the somite develop in place from embryonic through adult stages, becoming a thin epithelium that borders the somitocoel. Hereafter, we refer to the lateral somite cells as the external cell layer (Table 1; Figures 3, 4, and 5). Black arrowheads mark the transition between the dorsal myotome and external cell layer in embryonic through mid-larval stages (Figures 3A,B,E,F and 4A,B). In late larvae (Figure 4C), the process of a external cell can be see extending ventrally along the neural tube (black arrowhead); however, cell bodies were not observed to do so. By adult stages, the lateral mesothelium derived from the external cell layer is continuous with the sclerotome-derived mesothelium. Morphologically, the sclerotome and external cell layer-derived populations are indistinguishable, described further below. In adults, the mesothelium derived from the external cell layer forms the lateral boundary of the somitocoel and the muscle forms the medial boundary. Thus, the somitocoel remains in place throughout development (black arrows indicate somitocoel in Figures 3A,B,C,D,F and 4A,B,C). The extracellular collagen of the dermis (described below) forms lateral to the external cell layer.

#### C. Development of fin box from the dorsal-lateral somite (external cell layer)

Fin boxes are segmental structures that form dorsal to the neural tube (schematized in Figure 1). They consist of a mesothelial lining enclosing a coelom. Extracellular matrix accumulates in the dermis between the dorsal mesothelium and overlying epidermis, increasing the height of fin boxes [15,17]. Our developmental series indicates that the fin box mesothelium originates from the dorsal part of the external layer of the somites (Figure 6). In embryos and early larvae, the dorsal border between the myotome and the non-myotome somite is adjacent to the dorsal neural tube (Figures 3A,B and 6A, black arrowheads). Dorsally, the neural tube directly abuts the epidermis (Figure 6A). At the 4 GS stage, the dorsal-most cells of the external cell layer in each somite pair appear pinched medially toward each other (Figure 6B, black arrowheads). At the 5 GS stage (Figure 6C), a single layer of cells extends dorsally over the neural tube and remains continuous with the external cell layer of both somites. At the 5 to 6 GS stages, the mesoderm overlying the neural tube has become double layered and has lost contact with somites (Figure 6D). While the non-myotome somite cells have considerably thinned by this stage (black arrowhead), mesothelial cells in the fin box remain larger, similar to earlier stages (black arrow). Hemal fluid often accumulates around the fin box. By early metamorphosis, fin box coeloms expand (Figure 6E). Each fin box coelom is enclosed by mesothelial cells on all sides, evident when comparing transverse and sagittal views (Figure 6E,F). In a subadult (Figure 6G,H,I), fin box coeloms are further enlarged, and mesothelial cells are thin and flattened (black arrow), similar to the other somite-derived mesothelia described above. Extracellular material accumulates within the dermis between the dorsal mesothelium of the fin box coelom and the epidermis, and a file of cells extends between them along the dorsal midline (Figure 6G, white arrowhead). Such structures within the dermis have been previously described as cutaneous canals [17]. Their developmental origin is unknown. Granular extracellular material is densest surrounding them, suggesting that they may secrete it (Figure 6H,I).

### Development of amphioxus connective tissues

**Figure 7:**
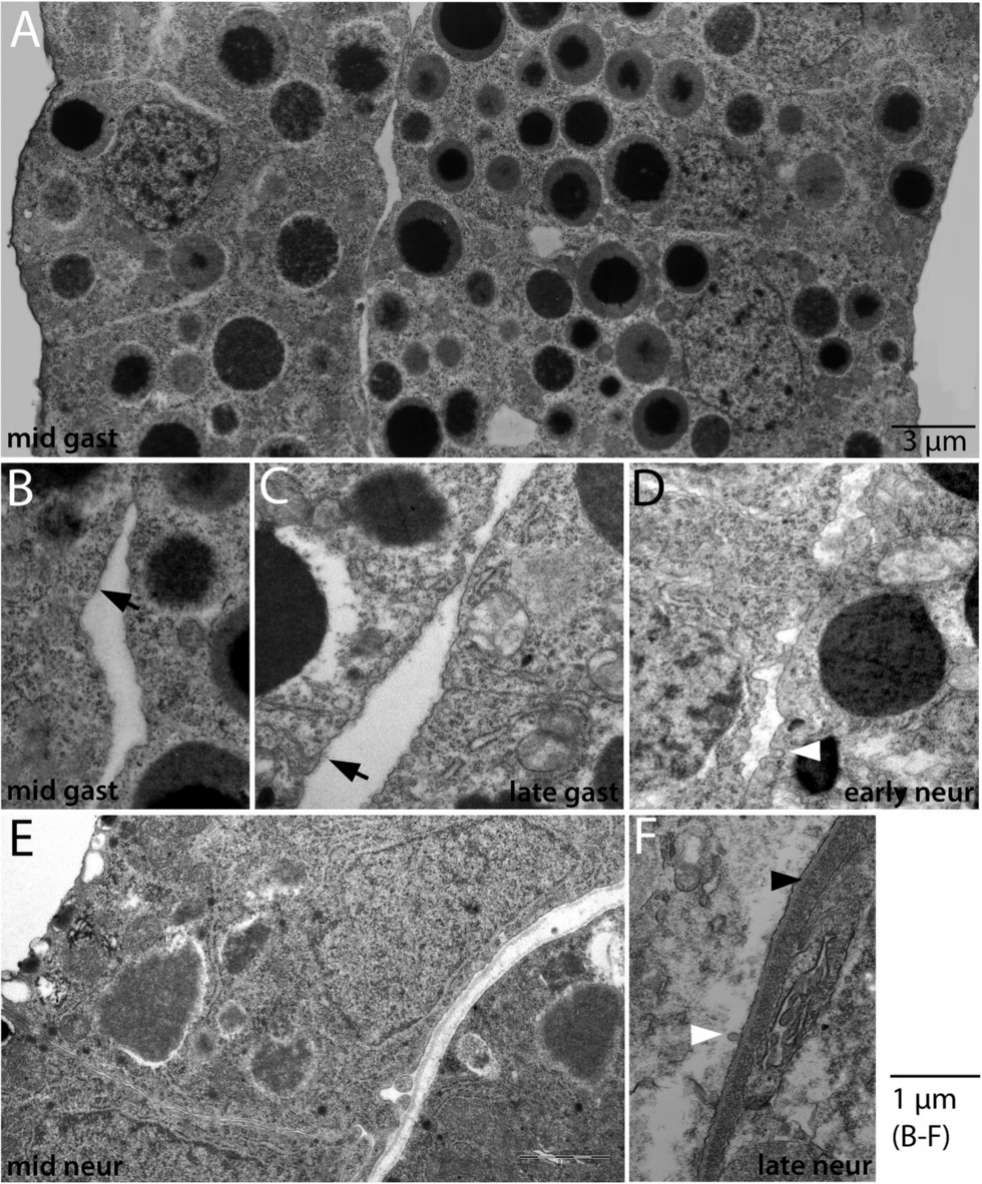
Development of the dermis (1): embryonic stages. Extracellular material giving rise to the dermis is shown accumulating between the epidermal and mesendodermal cells during embryonic stages. In all panels, ectoderm is to the left, mesendoderm **(A, B)** or somitic mesoderm in **(C-F)** is to the right. **(A)** shows an overview, and **(B)** is a detail showing particulate extracellular material associated with both cell layers (arrow indicates matrix outside the epidermal cell in **B, C**). White arrowheads in **(D)** and **(F)** indicate clathrin-coated vesicles observed in both epidermis and mesoderm. Black arrowhead in **(F)** indicates the basal lamina formed by the end of embryogenesis. Images are to scale except A (as indicated), and all sections are transverse; dorsal is up, lateral to the left and medial to the right. Stage abbreviations: gast, gastrula; neur, neurula.

**Figure 8:**
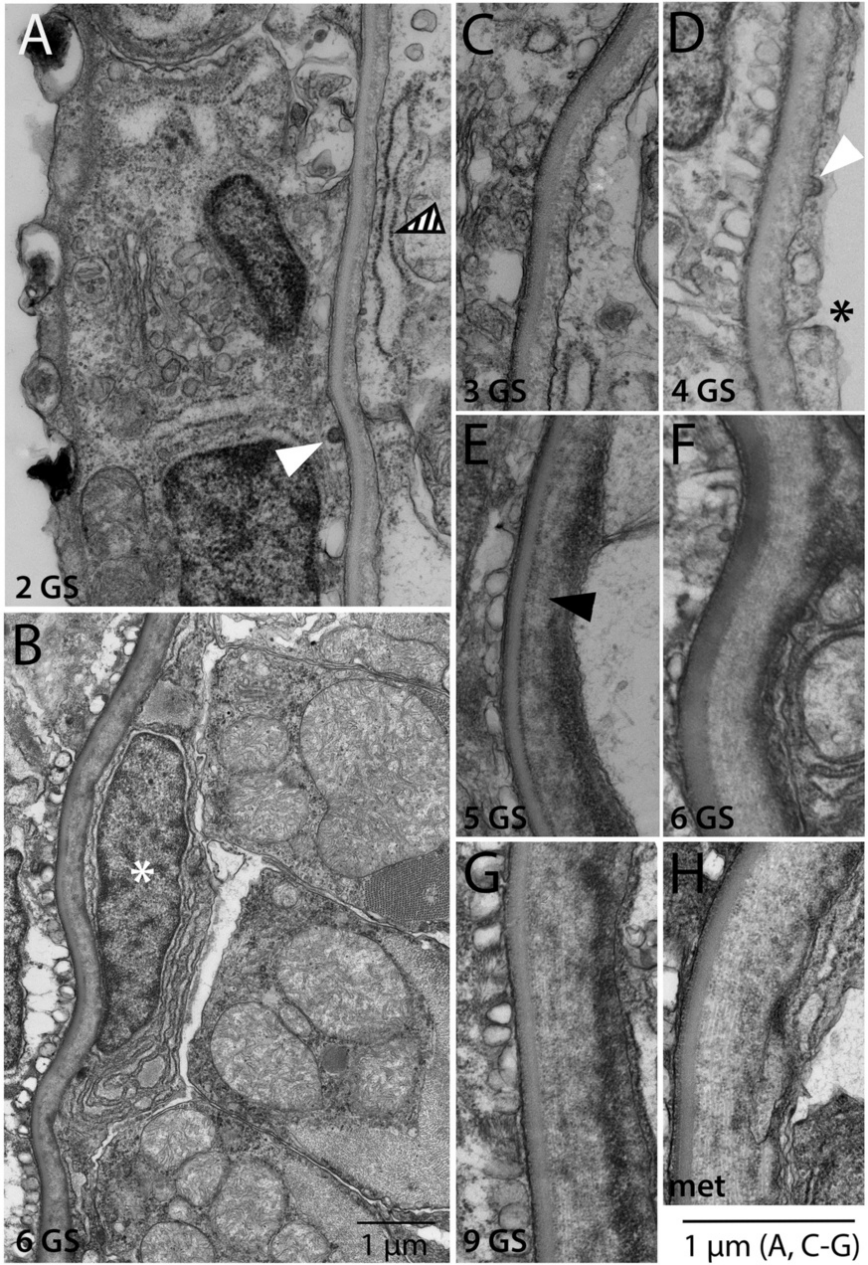
Development of the dermis (2): larval stages. Collagenous dermal extracellular matrix accumulates between the basal lamina of the epidermis and the underlying mesoderm cells through larval stages **(A-H)**. In all panels, epidermal ectoderm is to the left, somitic mesoderm/mesothelium is to the right, and the developing dermis is shown at the interface between them. **(A, B)** show overviews at the indicated stages. White asterisk marks the nucleus of a mesothelial cell derived from the external cell layer. **(F)** is a detail of **(B)** that shows the dermis to scale with the other panels. Black arrowhead in E indicates striated collagen fibers, which appear first during mid-larval stages. White arrowheads in **(A)** and **(D)** indicate clathrin-coated vesicles. Striped arrowhead in **(A)** shows rough endoplasmic reticulum in a mesothelial cell derived from the external cell layer. Images are to scale except **(B)**, as indicated, and all sections are transverse; dorsal is up, lateral is to the left and medial is to the right. Stage abbreviations: GS, gill slit; met, early metamorphosis.

**Figure 9:**
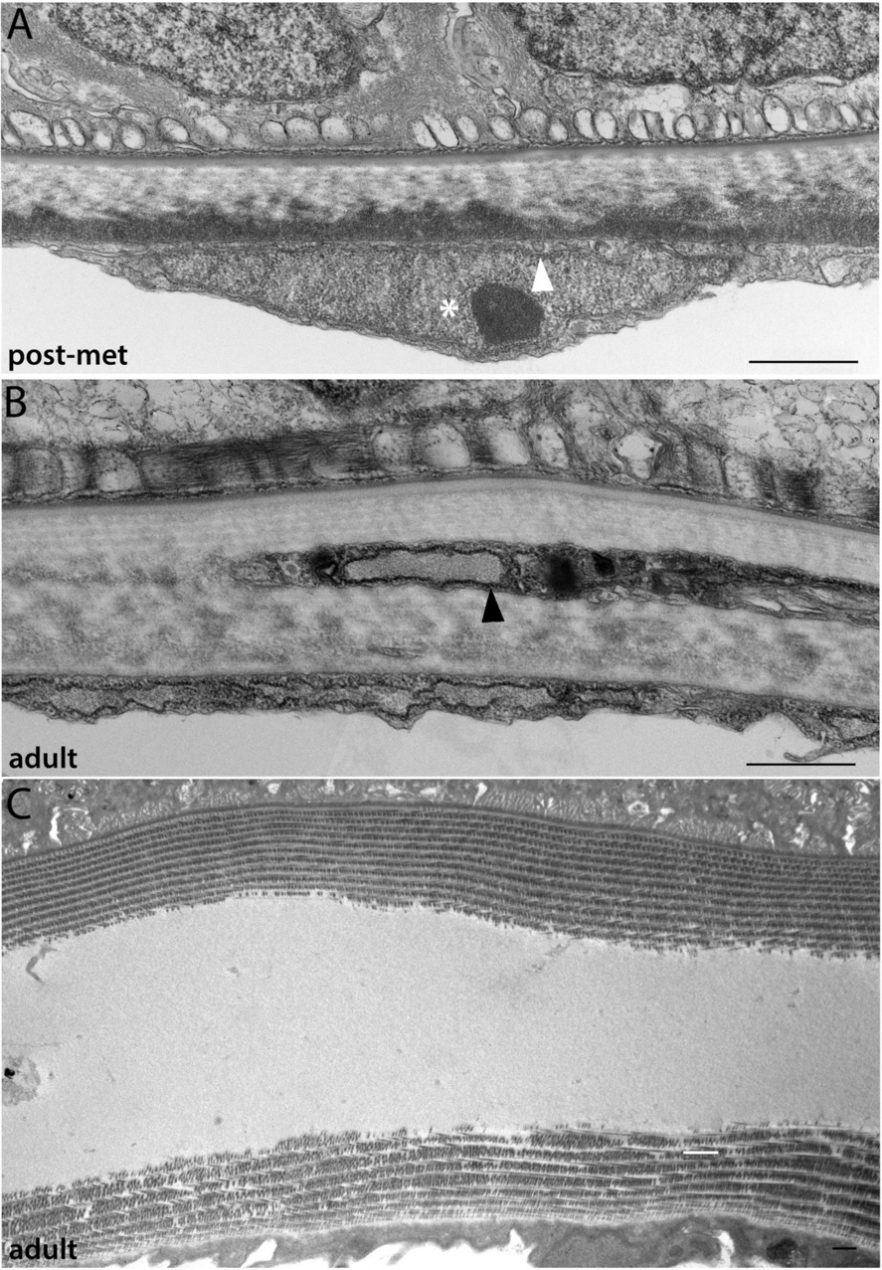
Development of the dermis (3): postmetamorphic and adult stages. **(A, B)** Striated collagen layers continue to increase within the dermis during juvenile and adult stages. In all panels, epidermal ectoderm is up and mesothelium derived from the external cell layer is down. White arrowhead marks a clathrin-coated vesicle; white asterisk marks the nucleus of a mesothelial cell derived from the external cell layer. Fibroblast-like cells appear embedded within the dermal collagen during adult stages (black arrowhead), which, like the mesothelial cells below them, are rich in rough ER. **(C)** In subadults, a gelatinous layer is sometimes observed between the dermis collagen layers. When cut in the sagittal plane in **(C)**, cross-hatching of the striated collagen is evident. A cutaneous canal within the gelatinous layer is visible on the left side of the panel. Scale bars are 1 μm. Stage abbreviations: post-met, post-metamorphic juvenile; adult, subadult.

**Figure 10:**
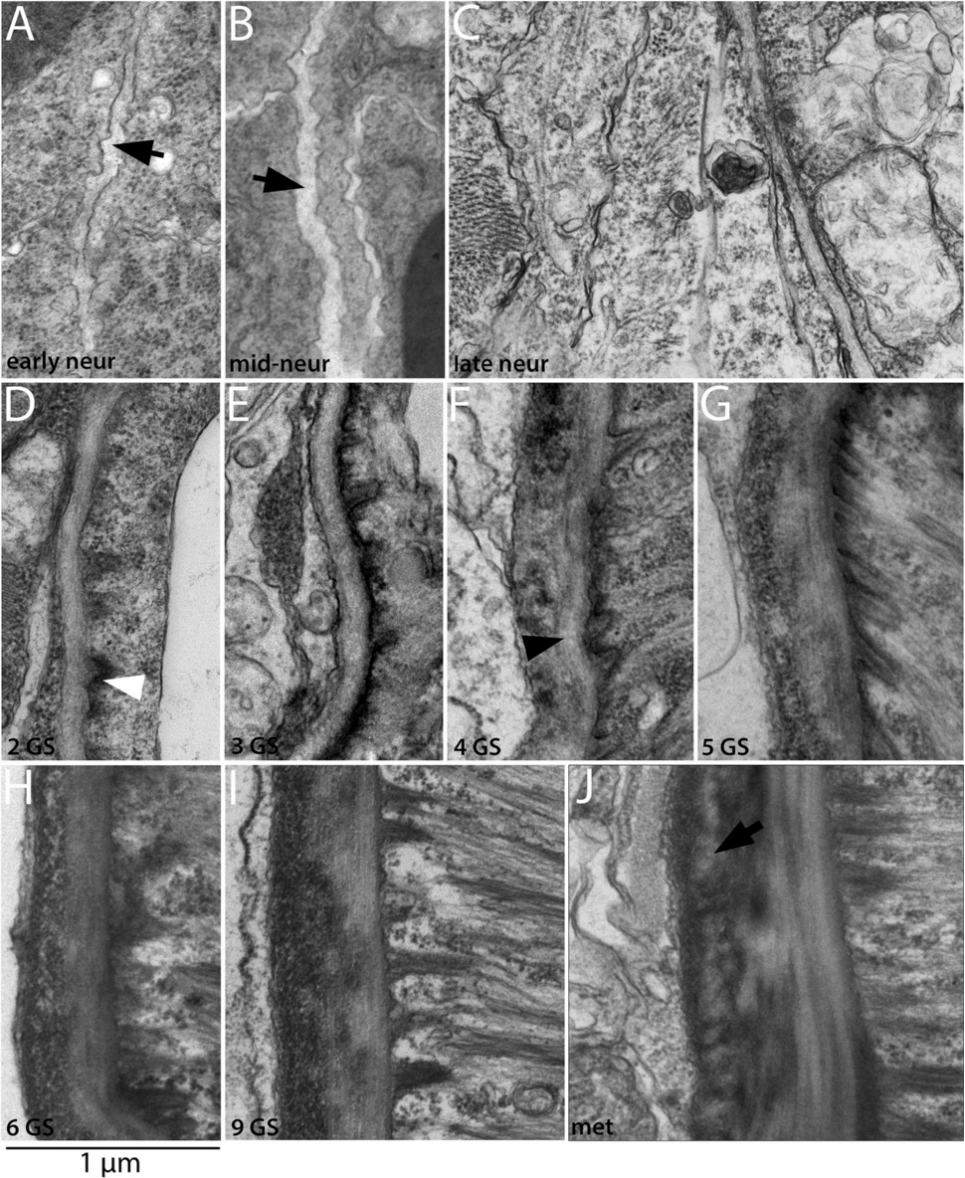
Development of the notochordal sheath (1): embryonic and larval stages. In all panels, the notochord is to the right and the somitic mesoderm is to the left (in panels **(A-E),** myotome is adjacent to the notochord, at later stages, in panels **(F-J)**, the sclerotome derived mesothelium is present between the notochord and myotome, see text). The notochordal sheath develops at the interface between them. Particulate extracellular matrix is present at the site of the future notochordal sheath in neurulae **(A-B)**, and a basal lamina associated with the notochord is present by late neurula stages **(C)**. Collagen increases within the sheath during larval and metamorphic stages **(D-J)**. White arrowhead indicates an anchor forming between the notochordal sheath and notochord lamella. Black arrowhead in **(F)** indicates striated (circumferential) collagen fibers, which are first apparent at mid-larval stages. Black arrow in **(J)** indicates the outer layer of longitudinal collagen fibers. All images are to scale and all sections are transverse. Stage abbreviations: neur, neurula; GS, gill slit; met, early metamorphosis.

**Figure 11:**
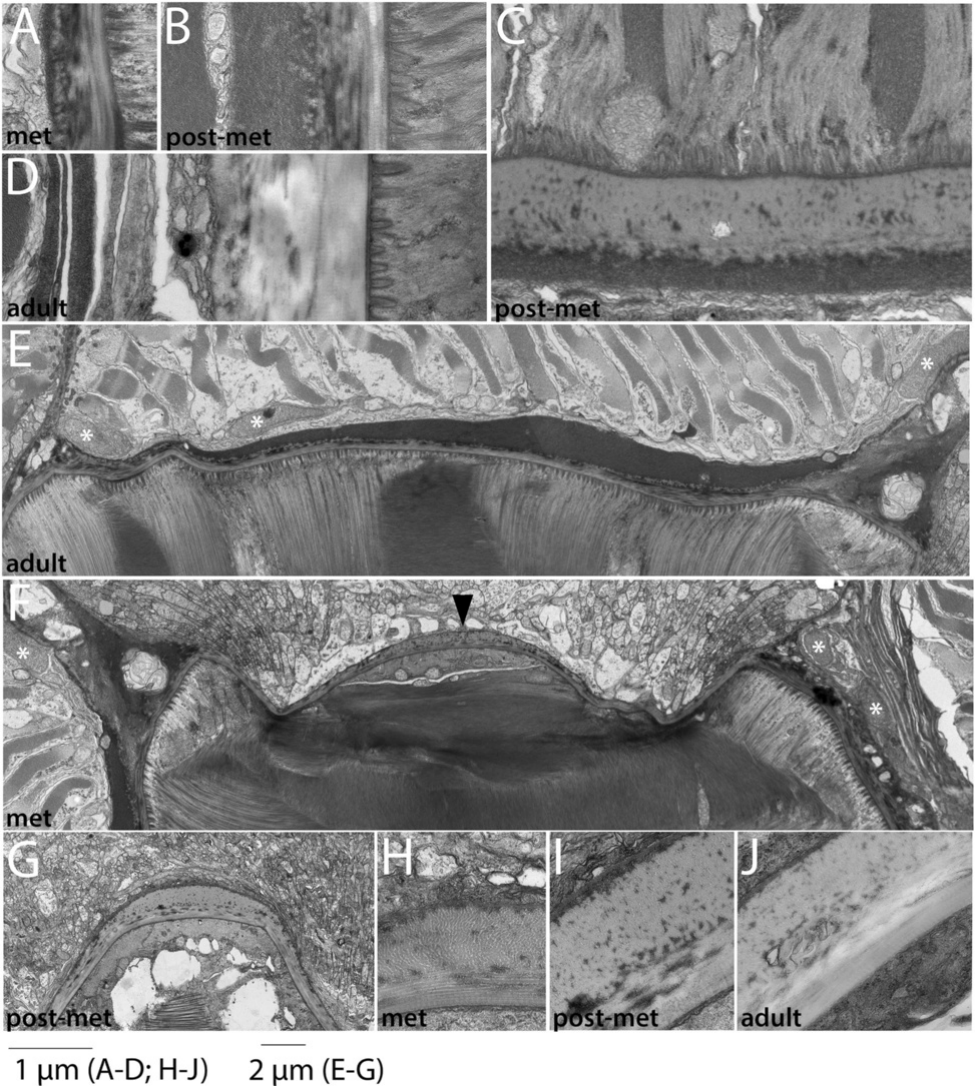
Development of the notochordal sheath (2): metamorphic and adult stages. Striated collagen layers are added to the notochordal sheath through metamorphic and into adult stages. (**A)** shows the same panel as in Figure 11J, now to scale with the sheath in postmetamorphic (**B)** and subadult **(C)** specimens. Notochord is to the right and the somitic mesoderm is to the left **(C)**. In a frontal plane, two individual notochord cells (top) can be observed terminating on the notochordal sheath (middle), and a sclerotome-derived mesothelial cell is seen at the bottom of the panel. Striated collagen fibers are cut in cross section in this view. (**E)** Overview of an entire side of the notochordal sheath in an early metamorphic animal; dorsal is to the right and the notochord is down. White asterisks indicate nuclei of sclerotome-derived mesothelium **(F, G).** Low power view of neural tube/notochord boundary, where there is a regional thickening of the collagen layers. In both panels, neural tube (dorsal) is up and notochord is down. White asterisks indicate nuclei of sclerotome-derived mesothelium. Black arrowhead indicates increased number of longitudinal collagen fibers in the notochordal sheath at the dorsal midline. **(H-J)** Details of the neural tube-notochord boundary at the dorsal midline. Panels **(A-D; H-J)** are to scale, and panels **(E-G)** are to scale, as indicated. Stage abbreviations: met, early metamorphosis; post-met, postmetamorphic juvenile; adult, subadult.

**Figure 12:**
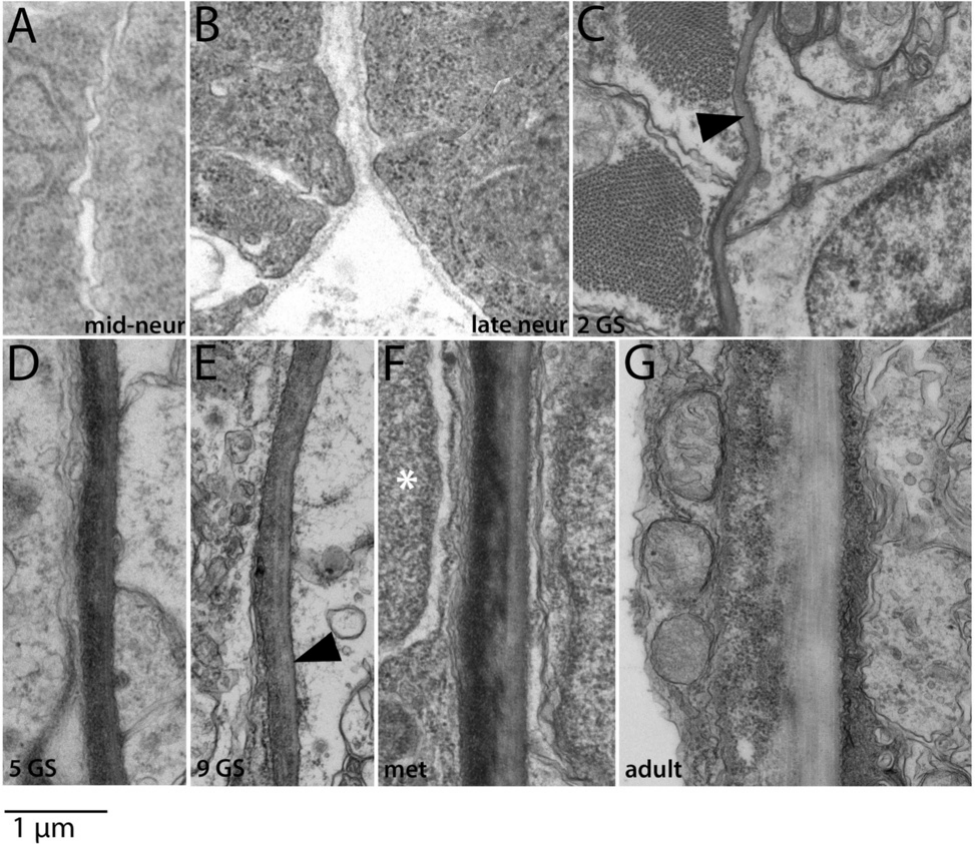
Development of the perineural sheath. In all panels, the neural tube is to the right. In panels **(A-E)**, myotome is adjacent to (left of) the perineural sheath, and in panels **(F-G)**, sclerotome-derived mesothelium is present between the myotome and perineural sheath (see text). The perineural sheath develops at the interface between them. A basal lamina is first apparent in 2 GS larvae **(C)**. The black arrowhead in **(E)** indicates striated collagen fibers, first apparent at the 9 GS stage, and the white asterisk in **(F)** marks the nucleus of a sclerotome-derived mesothelial cell. All panels are to the scale shown. Stage abbreviations: neur, neurula; GS, gill slit; met, early metamorphosis; adult, subadult.

**Figure 13:**
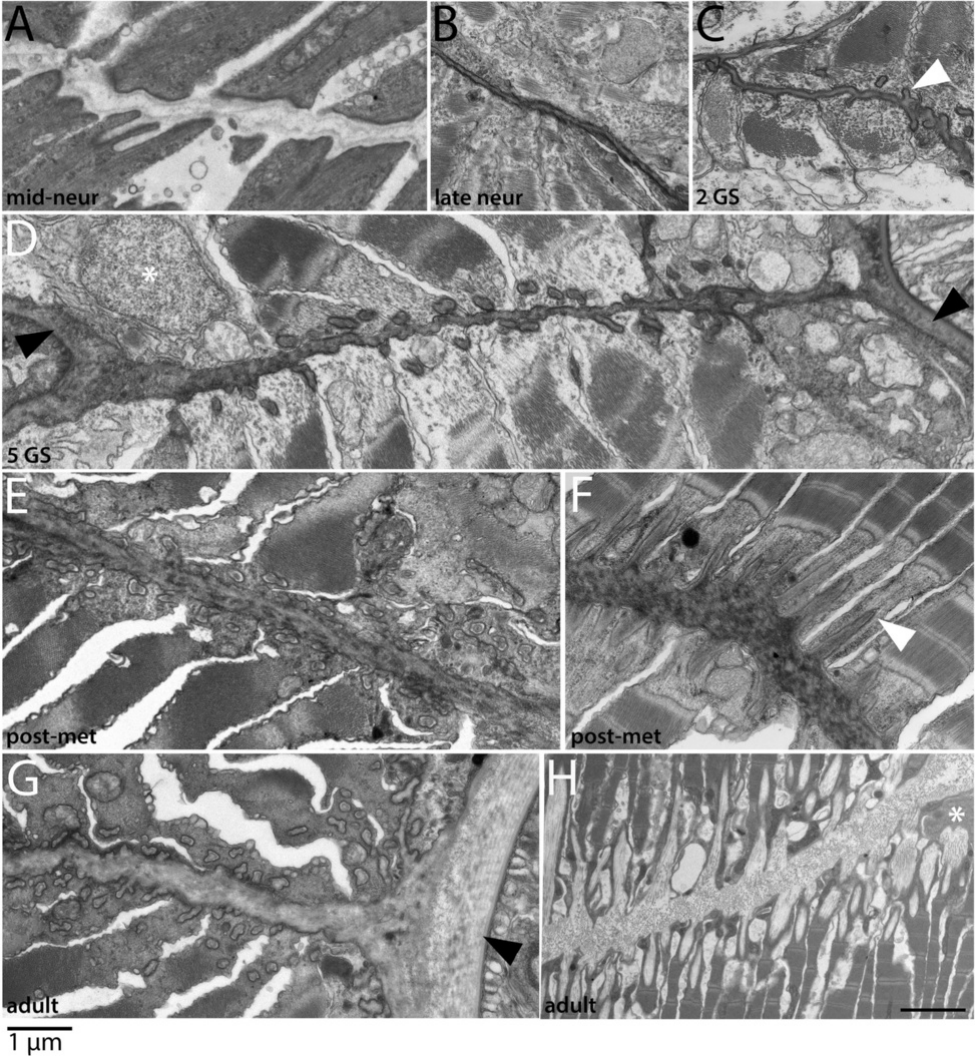
Development of the myosepta. In all panels, myosepta are shown forming between adjacent myomeres. Myosepta are shown in frontal section **(A),** in sagittal section (**F, H)** or in transverse section (remaining panels). **(A)** Extracellular matrix is evident in the position where myosepta form by mid-neurula stages, and **(B)** a double basal lamina is present in late neurulae. **(C)** In early larvae, anchors (white arrowhead) that attach muscle cells to the myosepta are first apparent. **(D)** A complete larval myoseptum is shown, attaching to the notochordal sheath (left) and the dermis (right); nucleus of a sclerotome-derived mesothelial cell is marked with a white asterisk. **(E-F)** Collagen accumulates within the myosepta during larval and metamorphic stages, although it does not appear striated. In sagittal section **(F)**, the specialized ends of the muscle cells where they terminate on myosepta, are evident. White arrowhead indicates an anchor extending into the muscle cell. **(G-H)** The myosepta are further thickened in subadults, shown in transverse and sagittal sections, respectively. Black arrowhead in **(G)** indicates the dermis, white asterisk in **(H)** indicates the nucleus of a myoseptal cell that appears late in development along the myosepta and is not a muscle cell (see text). All images are to the scale shown at bottom left except **(H)** where scale bar is 2 μm. Stage abbreviations: neur, neurula; GS, gill slit; post-met, postmetamorphic juvenile; adult, subadult.

**Figure 14:**
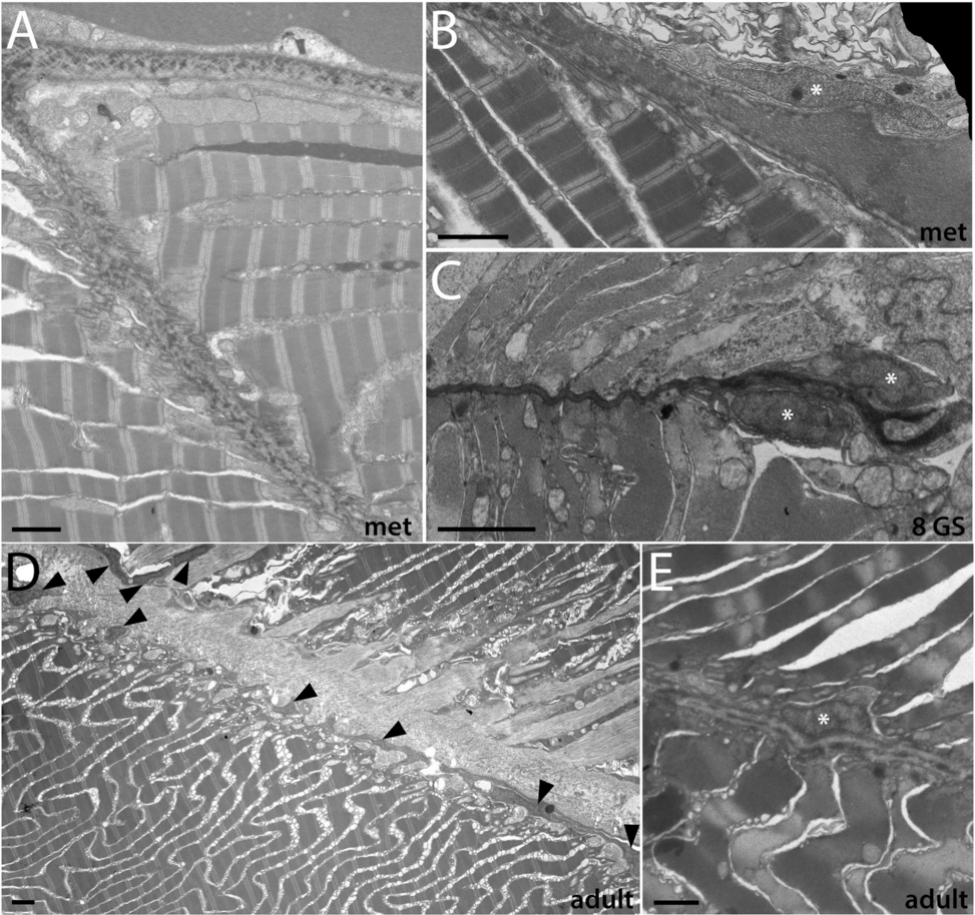
Cell migration into the myosepta. In late larval stages, fibroblast-like cells are rare but present along both sides of the myosepta, and these become abundant at adult stages. (**A)** Overview of metamorphic stage myoseptum, with no non-muscle cells present. Rarely, fibroblast-like non-muscle cells are observed within the myosepta in metamorphic **(B)** and late larval **(C)** stages, where they are most commonly found near the ends of the myosepta and continuous with the mesothelium derived from either the sclerotome or external cell layer. Nuclei of myoseptal cells are marked by asterisks. **(D)** In a subadult, fibroblast-like myoseptal cells are present along the length of each myoseptum (arrowheads indicate nuclei). **(E)** Detail of one myoseptal cell (nucleus marked by an asterisk). Panels **(A)**, **(B)**, and **(D)** are sagittal sections; **(C)** and **(E)** are transverse sections. Stage abbreviations: GS, gill slit; met, early metamorphosis; adult, subadult.

Amphioxus axial connective tissues consist of extracellular collagen layers underlain by basal laminae. Because we wished to characterize the development and fate of the non-myotome somite cells, including a possible role in producing these connective tissues, we next examined connective tissue ontogeny, shown in Figures 7, 8, 9, 10, 11, 12, 13, and 14.

#### A. Dermis

The adult dermis is schematized in Figure 1 and was described previously [17]. It consists of extracellular striated collagen beneath the epidermis and separated from it by a basal lamina. A non-striated, gelatinous layer of extracellular matrix is often found between layers of striated collagen. The dermis is bounded on its medial side by somite-derived structures: the external cell layer mesothelium (dorsally) or the perivisceral coelom mesothelium (ventrally).

We traced the origin and development of the dermis (Figures 7, 8, and 9). In mid-gastrulae, the interface between the ectoderm and mesendoderm (where the dermis will form) contains some particulate extracellular matrix (Figure 7A,B, arrow) that continues to accumulate during late gastrula through mid-neurula stages (Figure 7C (arrow),D,E). The material appears along both sides of the interface (slightly pulled apart in the preparations shown), thus it may be produced by cells in both layers. Indeed, coated vesicles filled with ECM and opening to the extracellular space are observed on both sides of the membrane, although it cannot be determined from a static image whether these are exocytotic or endocytotic vesicles (Figure 7D,F, white arrowheads). A basal lamina associated with the ectoderm is first distinguishable in late neurulae (Figure 7F, black arrowhead). During early larval stages, granular extracellular material of the dermis continues to increase in thickness (Figure 8A,B,C,D). Striated collagen fibers are first apparent around the 5 GS stage (Figure 8E, arrowhead), and increase during late larval stages (Figure 8F,G). In mid-late larvae, hemal fluid accumulates medial to the collagen and intermixes with collagen layers. The number of striated collagen layers continues to increase during metamorphic, juvenile and adult stages, reaching an apparent average thickness of about 1.5 μm in a subadult (Figures 8H and 9A,B). In postmetamorphic stages, we observed cells embedded within the dermal collagen (Figure 9B, arrowhead). These cells may be fibroblasts previously described [17,39]; their developmental origin is unknown but discussed further below. The crosshatched structure of dermal collagen fibers is evident in sagittal sections at postmetamorphic stages, and an expanded gelatinous layer is sometimes observed between layers of collagen fibers in subadults (Figure 9C and [17]). Both the ectoderm and the underlying somite-derived mesothelium remain closely associated with the dermis throughout its development.

#### B. Notochordal sheath

The notochordal sheath (schematized in Figure 1) consists of three layers of extracellular material [17,40-42]. The outermost layer is composed of longitudinal, striated collagen fibers, the middle layer of circumferential (radial) striated collagen, and the inner layer, also called the elastic interna, is a basal lamina. The notochordal sheath borders the perineural sheath dorsally, the gut tube ventrally and the mesothelia derived from the sclerotome on either side. We find that the sequence and timing of its development is similar to that of the dermis.

The origin and development of the notochordal sheath is shown in Figures 10 and 11. The notochord pinches off from paraxial mesoderm during early neurulation, and immediately after their separation, particulate extracellular matrix is observed between the notochord and somites (Figure 10A,B, arrows). In late neurulae, a basal lamina is present around the notochord (Figure 10C). During early larval stages, extracellular material accumulates between the notochord basal lamina and the adjacent myotome cells. The innermost layer of the notochordal sheath attaches to the notochord via anchors containing hemidesmosome junctions [17]. These anchors are first apparent around the 2 GS stage (Figure 10D, white arrowhead) and become increasingly more developed during the larval period (Figure 10E,F,G,H,I). The proposal that such anchors are cilia-related structures [43] is incorrect. Between the 4 and 5 GS stages, striated, circumferential collagen fibers first become apparent within the sheath (Figure 10F, black arrowhead). The outer layer of longitudinal fibers is thinner and develops later; we first observed it during metamorphosis (Figures 10J, arrow, and 12A,B,C,D). The outer fibers of the sheath (both longitudinal and circumferential) often become intermixed with hemal fluid. Layers of striated collagen continue to be added during the metamorphic and juvenile stages (Figure 11A,B,C,E) and reach a thickness of approximately 1.5 μm (similar to the dermis) in a subadult (Figure 11D). A previous study reported that the adult notochordal sheath can reach a thickness of at least 20 μm [17], indicating that growth continues in adulthood. At the dorsal midline, the notochordal sheath merges with the perineural sheath and the outer, longitudinal collagen layers are thicker than elsewhere (Figure 11F (arrowhead),G,H,I,J). At this position, the sheath is bounded dorsally by the floor plate and ventrally by specialized notochord cells called Müller cells (Figure 11F). The number of layers of these longitudinal collagen fibers increase during metamorphosis and juvenile stages and reached a thickness of about 2 μm in a subadult (Figure 11J).

#### C. Perineural sheath

The perineural sheath (schematized in Figure 1) consists of circumferentially oriented collagen fibers underlain by a basal lamina. It borders the sclerotome-derived mesothelium on either side and merges with the notochordal sheath ventrally. Dorsally, it is adjacent to the epidermis, fin box, and finally the external cell layer-derived mesothelium at different stages of development. It is thinner than the notochordal sheath and dermis, and its development is delayed relative to these structures.

Perineural sheath development is shown in Figure 12. During its formation, the neural tube is flanked by the paraxial mesoderm and borders the axial mesoderm ventrally. The early stages of perineural sheath formation are identical to that of the notochordal sheath and dermis. Particulate extracellular material accumulates on both sides of the interface between the neural tube and somites by the late neurula stage (Figure 12A,B), and a basal lamina appears around the neural tube in early larvae (Figure 12C). The perineural sheath grows during larval stages through addition of particulate extracellular matrix. Unlike in the dermis and notochordal sheath, striated collagen fibers are not present in the perineural sheath at the 5 GS stage (Figure 12D); rather, they first appear in late larvae, around the 9 GS stage (Figure 12E). The perineural sheath increases in thickness during metamorphic (Figure 12F) and juvenile stages and is about 0.75 μm in a subadult (Figure 12G), or about half the thickness of the dermis and notochordal sheaths.

#### D. Myosepta

The myosepta are composed of collagenous extracellular material and form between the anterior and posterior borders of adjacent segments (schematized in Figure 1). The myosepta connect laterally to the dermis and medially to the notochordal sheath and transmit force from contracting muscles to the notochord. In contrast to vertebrates, amphioxus axial muscles never connect directly to the notochordal sheath or other midline structures; their only tendinous connection is to myosepta [44].

Myoseptal development is shown in Figures 13 and 14. In mid-neurulae, myosepta consist of a layer of extracellular matrix at the interface between adjacent myotomes (Figure 13A), and in late neurulae develop into double basal laminae facing both myotomes (Figure 3B). The myosepta increase in thickness during larval and metamorphic stages through addition of particular extracellular material, although striated collagen fibers are not observed (Figure 13C,D,E,F). In subadults, the myosepta reach a variable thickness of 0.5 to 1 μm (Figure 13G,H). Figure 13D shows a complete myoseptum at the 5 GS stage, including its attachments to the notochordal sheath and dermis (left and right arrowheads, respectively). An attachment to the (now striated) dermis is also shown for a postmetamorphic juvenile (Figure 13G, black arrowhead). The myosepta attach to muscle cells through subcellular structures termed microtendons [17], which are evident by early larval stages (Figure 13C, white arrowhead) and continue to develop in larvae and juveniles. In transverse sections (Figure 14C,D,E,G), the microtendons are largely perpendicular to the section plane, so their connections to the myosepta are mostly not visible; in sagittal sections (Figure 13F,H), these connections are more easily visualized. Myofibers become excluded from the microtendon forming regions of muscle cells in postmetamorphic stages, evident in sagittal section (Figure 13F).

The myosepta develop between muscle cells of adjacent segments and are therefore likely produced by muscles themselves. However, in adult animals, we observed non-muscle cells beside the myosepta as well (Figure 13H, asterisk marks a nucleus). To further characterize these myoseptal cells, we examined multiple stages. They are not present in embryos or early larvae and are rare in late larvae. For example, a sagittal section through a metamorphic stage myoseptum reveals no non-muscle cells (Figure 14A). When these cells are observed in late larvae and metamorphic animals (Figure 14B,C, nuclei marked by asterisks), they are usually but not always near the ends of the myosepta and in contact with either the sclerotome or external cell layer-derived mesothelium. In contrast, in adults, non-muscle cells are found along the length of myosepta (Figure 14D, E). The possibility that these cells are somite-derived is considered below.

### Connective tissue fate of non-myotome somite cells

All of the non-myotome somite derivatives described above develop adjacent to extracellular connective tissues. We therefore wished to ask whether they show features of secretory cells that would suggest they contribute to these connective tissues.

For this analysis, we first referred back to high magnification views of individual cells in the TEM series (Figures 3, 4, 5, 6, 7, 8, 9, 10, 11, 12, 13, and 14). Morphologically, all non-myotome somite cells and their mesothelial derivatives in the fin box, external cell layer and sclerotome (and perivisceral coelom, not shown), are indistinguishable at the stages we examined, although some ultrastructural differences have been noted in adults, notably in the presence of cilia [15]. In embryos and early larvae, non-myotome somite cells form a circumferentially oriented epithelium continuous with myotome (Figure 3E,F,G,H,I, asterisks mark nuclei). During larval stages, these cells remain epithelial and become elongated and extremely thin; their thickest point is often their nuclei (Figures 4C and 5A show overviews, and higher power views are shown in Figures 8A,B, 9A,B, 4D,E,F,G,H,I, and 5B,C,D, nuclei marked with white asterisks). Adherens junctions are observed between them (for example, Figures 5E and 8D, black asterisks), indicating that they remain epithelial. These cells are uniformly rich in rough endoplasmic reticulum from larval through adult stages, in agreement with previous findings in adults [15], and suggesting that they are specialized for protein secretion (one example of rough ER is indicated by the striped arrowhead in Figure 8A, but see also Figures 3, 4, 5, 8, and 9, especially Figures 3I, 4F,G,H,I, 5B,C,D, 8A,B, and 9B). Further, we observed vesicles, including clathrin-coated vesicles, opening between these cells and extracellular collagen layers (for example, Figures 7D, 8D, and 9A, white arrowheads). However, based on static images, it cannot be determined whether these vesicles are exocytotic or endocytotic, and it is also is important to note that vesicles were also observed in the ectodermal epithelium, neurons, and notochord (for example, Figures 7F and 8A, white arrowheads).

**Figure 15:**
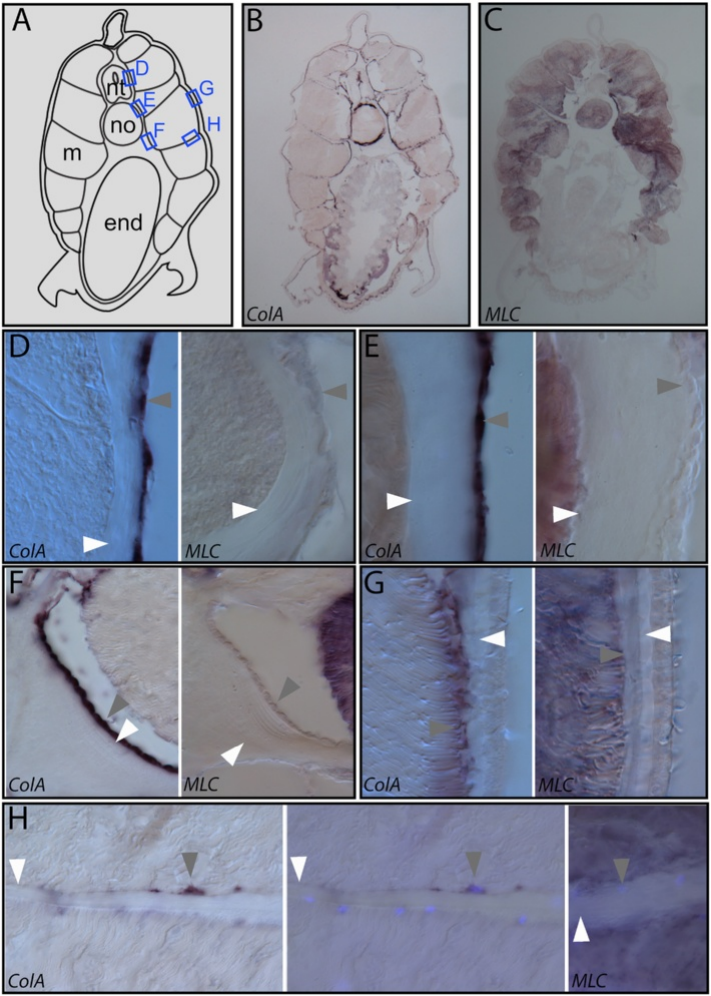
*Collagen A* is expressed in the non-myotome somite derivatives in adults. In adults, all derivatives of the non-myotome somite cells are directly apposed to extracellular collagen layers and express the fibrillar collagen gene *ColA*, suggesting a connective tissue identity. **(A)** Schematic of transverse sections shown in **(B, C)**; boxes indicate positions detailed in panels **(D-H). (B, C)** Adjacent transverse sections show expression of *collagen A* (*ColA*) and *Myosin Light Chain* (*MLC*), respectively. *MLC* marks the myotome and notochord cells, and *ColA* expression is observed the mesothelia that line the collagen layers including the perineural sheath, notochordal sheath, dermis, fin box coelom, and myosepta. Details in **(D-H)** show adjacent sections stained with *ColA* or *MLC* from regions boxed in **(A)**. White arrowheads indicate extracellular collagen layers, and grey arrowheads indicate *ColA*-expressing mesothelial cells derived from somites. **(D)** Perineural sheath and the sclerotome-derived mesothelium **(E)** notochordal sheath and the sclerotome-derived mesothelium **(F)** notochord/gut boundary, position where the notochordal sheath collagen is thickened, and the sclerotome-derived mesothelium **(G)** dermis and the external cell layer-derived mesothelium. (H) Myoseptum with myoseptal cells lining it. Left panel shows brightfield; middle panel overlays a DAPI image to show nuclei of the fibroblast-like myoseptal cells. Myoseptal cells strongly express collagen (left and middle panels) but not *MLC* (right panel, which includes DAPI overlay), while the surrounding muscle stains for *MLC* (right panel). Note that muscle cell nuclei located laterally and are not found along myosepta, thus are not visible in these panels. In all panels, dorsal is up, medial is to the left. Micrographs were taken at 200× **(B, C)** or 1,200× **(G, H)** magnification. Abbreviations: end, endoderm; m, muscle; no, notochord; nt, neural tube.

**Figure 16:**
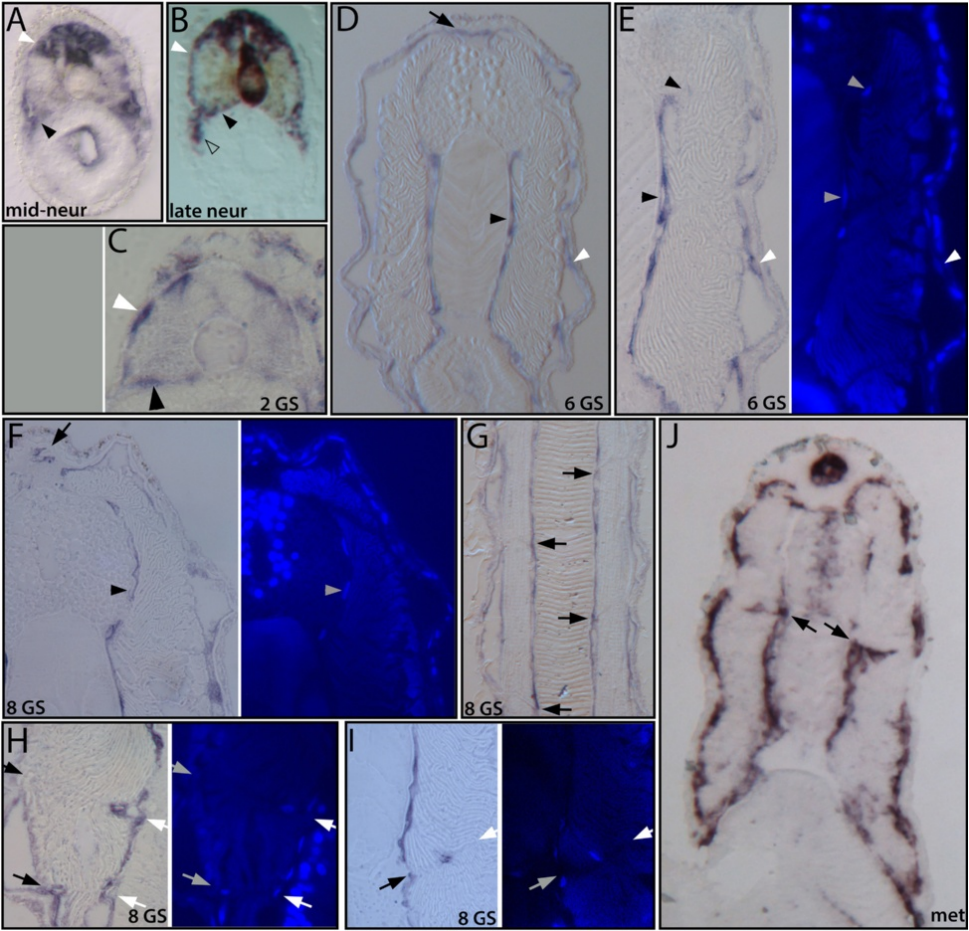
*Collage A* is expressed in non-myotome somite cells throughout development. In each panel, black arrowheads indicate sclerotome mesothelium and white arrowheads indicate the external cell layer mesothelium. **(A, B)** During embryonic stages, non-myotome cells express *ColA.* Expression does not overlap with *MLC*, which marks myotome, stained brown in the double-labeled embryo in panel **(B)**. During development, the position of *ColA* expressing cells tracks the position of sclerotome and external cell layer derivatives observed by TEM (see text). **(C)** and **(D)** show overviews, **(E)** shows a detail of **(D)** in brightfield (left) and DAPI nuclear stain (right). Arrowheads mark the same nuclei in **(D-E)**; arrow marks the fin box mesothelium. **(F)** In later larvae, the sclerotome mesothelium extends to the neural tube level and continues to express *ColA* (brightfield, left panel; DAPI, right panel), as does the external cell layer and fin box mesothelium (arrow). **(G)** A frontal section shows no variation in morphology or gene expression along the anterior-posterior axis of segments (black arrows mark the medial ends of the myosepta). **(H-I)** In late larvae, non-muscle cells are seen within the myosepta. These are often adjacent to the mesothelium of the external cell layer or the sclerotome **(H)**, but were sometimes observed in the middle of myosepta **(I)**. Brightfield (left) and DAPI (right) panels are shown; arrows mark the medial (black) and lateral (white) ends of the myosepta. (**J)** Overview of an early metamorphic stage. The mesothelia derived from sclerotome and external cell layer, the fin box and myosepta are all *ColA* positive. Black arrows mark the medial ends of myosepta; labeled myoseptal cells are connected to the sclerotome mesothelium similar to **(H)**. All sections are transverse except **(G)**, which is frontal. Micrographs were taken at 1,200× (A-G) or 1,000× **(J)** magnification.

We also examined the expression of *collagen A* in our developmental series (Figures 15 and 16). This is the single clade A collagen gene in amphioxus. Clade A family members in vertebrates (including collagens 1, 3, 5, and 2) form the predominant fibrillar components of connective and skeletal tissues including dermis, tendon, ligament, cartilage, and bone [45]. The *ColA* gene was previously shown to be expressed in somites, notochord, and neural tube of amphioxus embryos, [36,46].

In adults, we find that *ColA* is strongly expressed in all of the somite-derived mesothelia described above, including the external cell layer and sclerotome mesothelia, and the mesothelia of the fin box and perivisceral coelom (Figure 15B,D,E,F,G). It is also strongly expressed in the non-muscle cells that become abundant in the myosepta in adults (Figure 15H). Finally, *ColA* is expressed in the neural tube and the Müller cells, which are located on the dorsal and ventral periphery of the notochord (Figure 15B). Adjacent sections were stained with a muscle myosin light chain probe (*MLC*), which labels the myotome and also the notochord lamellae, which are contractile in amphioxus, unlike in vertebrates [17,41]. Unlike the non-myotome somite derivatives, *ColA* mRNA was never detected in myotome cells (Figures 15 and 16).

We next observed *ColA* expression in our developmental series, and we found that it is expressed in the non-myotome somite and its derivatives throughout development. In embryos, *ColA* is expressed in all, or nearly all, cells of the non-myotome somite epithelium (Figure 16A,B, white arrowheads indicate the external cell layer, black arrowheads indicate sclerotome). Double *in situ* hybridization with *MLC* shows no overlap between the two markers in somites (Figure 16B). Consistent with prior reports, the embryonic notochord and neural tube express *ColA* as well [36,46]. However, expression by these midline structures is labile, and during early larval stages, both tissues extinguish *ColA* expression (Figure 16D,G,I), which then resumes later in the neural tube and in a the notochord Müller cells (Figure 15B, described above).

Strikingly, in larvae, *ColA* expression continues strongly in all derivatives of the non-myotome somites. Tracking the position of *ColA* positive cells reveals they are present in all the same positions where we observed sclerotome-derived mesothelium (Figure 16D,E,F,G,H,I,J). Frontal (horizontal) sections show no variation in the position of sclerotome-derived mesothelial cells along the AP axis of each segment (Figure 16G, and not shown), although at most time points we focused on transverse sections. In addition to expression in sclerotome derivatives, *ColA* expression is observed in the mesothelium derived from the external cell layer (Figure 16A,B,C,D,E,F,G,H,I,J, white arrowheads indicate selected nuclei) and in their derivatives including the lateral mesothelium and the fin box mesothelium (Figure 16D,F (black arrows),J). Finally, the non-muscle cells that populate the myosepta also strongly express *ColA*. As described above, these cells are first apparent in late larval stages, where they are most often found at the ends of myosepta and appear connected to the external cell layer or sclerotome mesothelium (Figure 16H,I,J, black and white arrows mark the medial and lateral ends of myosepta, respectively). However, occasionally *ColA* positive cells also appear near the center of a myoseptum (Figure 16I). In adults, as described above, these cells are abundant along each myoseptum (Figure 15D), and these strongly express *ColA* (Figure 15B,H). Together, the ultrastructure and *ColA* expression of all non-myotome somite derivatives throughout development suggests that they contribute to formation of extracellular collagen connective tissues to which they are directly apposed.

## Discussion

### The development and derivatives of amphioxus non-myotome somite cells

**Figure 17:**
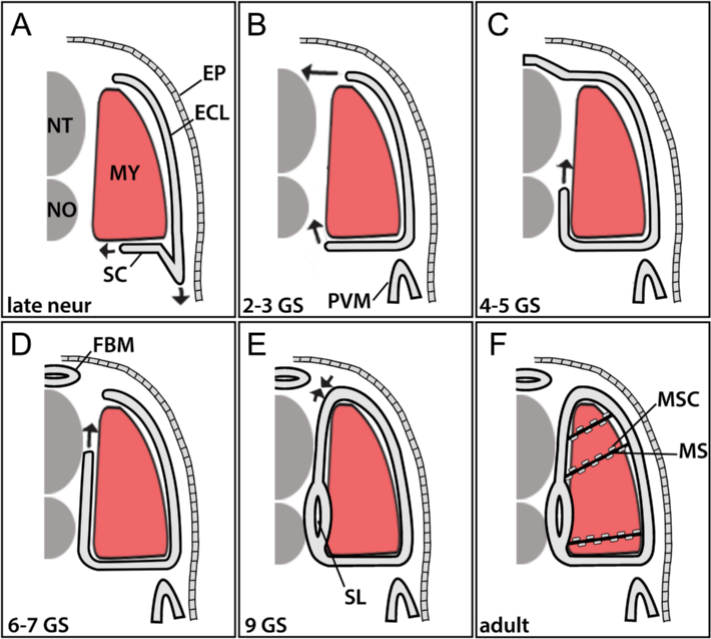
Summary of the development of amphioxus non-myotome somite derivatives in embryonic **(A)**, larval **(B-E)** and adult **(F)** stages. Note that arrows indicate a change in the relative position of tissues, but it is not known whether this is achieved by active migration and/or differential growth of somitic or surrounding tissues. See the text for a detailed description of development. Abbreviations: ECL, external cell layer; EP, ectodermal epithelium; FBM, fin box mesothelium; MS, myoseptum; MSC, myoseptal fibroblast cell MY, myotome: NT, neural tube; NO, notochord. Stage abbreviations: neur, neurula; GS, gill slit; post-met, post-metamorphic juvenile.

Our developmental series indicates that the non-myotome cells of amphioxus somites give rise to the mesothelia surrounding the muscle blocks, the mesothelial linings of the fin box and perivisceral coeloms, and likely to the fibroblast-like mesenchymal cells that are abundant along the myosepta between segments. Our series shows cell positions and apparent movements over time and is schematized in Figure 17.

The lateral part of the somite, the external cell layer, differentiates in place, as was previously reported, giving rise to the mesothelium that underlies the dermis. In contrast, the ventral somite, the sclerotome, undergoes considerable changes in cell position over time. It is important to note that we cannot determine that sclerotome cells are actively migrating, only that a relative change in position occurs, which could be due to migration and/or differential growth of sclerotome or the surrounding tissues. Previous conflicting models have proposed that the amphioxus sclerotome originates (1) from a diverticulum of ventral somite cells that move dorsally to surround the midline structures [27] or (2) from a population of dorsal somite cells that migrate ventrally along the midline [17]. Our developmental series indicates sclerotome cells originate from the ventral somite and move first medially toward the midline during late embryonic stages (Figure 17A). Simultaneously, the ventral-lateral somite pinches ventrally to give rise to the perivisceral coelom. During early-mid larval stages, the sclerotome is a single-layered mesothelium that moves progressively more dorsally along the midline structures and ultimately joins with the external cell layer at the level of the dorsal neural tube, to form a continuous mesothelium (Figure 17B,C,D,E). In contrast to Hatschek’s model, however, sclerotome cells are a single epithelial layer. It is only at the late larval stage that the mesothelium becomes double layered at the level of the notochord (Figure 17E), and it apparently remains single layered beside the neural tube at least until the subadult stage (the latest stage we examined). A previous report found a double layer in older adults [15]. It is not clear how this double layer forms since we did not observe any intermediate stages. It could form by delamination of the single-layered sclerotome. Alternatively, it is it is possible that after the sclerotome migrates it evaginates medially and pinches off to form a sclerocoel; if so, then the sclerocoel would be derived from the original somitocoel.

The fin box mesothelium also originates from the non-myotome somite, specifically from the dorsal-most region of the external cell layer. These cells move medially, to form a mesothelium overlying the neural tube (Figure 17A,B). As with the sclerotome, it appears single layered initially and connected to the somites on either side of it. Subsequently, these cells apparently detach from the somites and form a double layer (Figure 17C), which encloses the coelom of the fin box. As with the sclerocoel, we did not observe intermediate stages, so it is not clear how a double layer arises; two possibilities include delamination of the single layer or dorsal evagination of a single cell layer that then pinches off from the somites.

Cells associated with the myosepta have not been previously described in amphioxus to our knowledge. These fibroblast-like cells appear late in larval stages (not shown in Figure 17 schematic) but only become abundant in adults, where they are found along the length of both sides of each myoseptum (Figure 17F). These cells may also be derived from non-myotome somites, because they first appear (during mid-larval stages) close to and usually connected to the mesothelia derived from sclerotome or external cell layer. In adults, they appear to be mesenchymal, and they express *ColA*, but not a muscle marker (*MLC*), like the other non-myotome somite derivatives. Notably, similar myoseptal cells are found in vertebrates including teleosts (discussed below), where they are known to originate in sclerotome. Finally, in amphioxus, similar cells resembling fibroblasts have been observed embedded in dermal collagen layers ([17,22,39] and see Figure 9B). It is possible that these dermal fibroblasts also have a somitic origin in amphioxus, perhaps arising from the external cell layer-derived mesothelium.

A limitation to note is that we focused mainly on transverse section planes and thus could have missed variation, particularly across the A-P extent of the segments. To account for this, we observed multiple sections and specimens, and we prepared some specimens (but not at all stages) in frontal or sagittal planes (some of which are shown above).

### A connective tissue identity for non-myotome somite cells

Our observations suggest that the non-myotome cells of amphioxus somites comprise a population of connective tissue progenitors. They express *ColA* throughout development and develop directly apposed to the extracellular, collagenous axial connective tissues of amphioxus: the notochordal and perineural sheaths and the dermis. The ultrastructure of these cells, most notably their abundance of rough endoplasmic reticulum, further supports the idea that they are specialized for protein secretion. Finally, differences have been described in the extracellular matrix within the somitocoel and sclerocoel and in the morphology of the lateral and medial mesothelial in adults (presence *vs*. absence of cilia; [15]), indicating that these mesothelia are sub-functionalized to some extent. However, in most respects, these mesothelia are indistinguishable in morphology and ultrastructure.

The amphioxus axial connective tissues are likely produced by multiple cell types, and relative contributions may vary at different developmental stages. The notochordal and perineural sheaths must be secreted at least in part by the notochord and neural tube cells themselves, because these are the only collagen-expressing adjacent tissues during their early development ([36,46,47] and see Figure 16). Both, however, express it dynamically, and it is the somite-derived mesothelial cells that are the predominant *ColA* expressing cells from mid-larval stages onward, and persisting in adults. We therefore hypothesize that a key function of these somite-derived mesothelia is to expand and maintain the connective tissues that the midline structures first generate. One notable exception is the notochord Müller cells, which do strongly express *ColA* in adults. The Müller cells are distinct from the main notochord lamellae, and their functions are not understood [17,41]. They express *ColA* in precisely the same positions where the notochordal sheath is not contacted by somite-derived mesothelium. Thus, in adults, the notochordal sheath is continuously bounded by *ColA* expressing cells but from two different sources (Figure 15B). A regional thickening at the ventral side correlates to where it is bounded by both the Müller cells and the sclerocoel, and the dorsal sheath thickens where it contacts the dorsal Müller cells and the neural tube. In contrast, *ColA* was never detected in epidermis, suggesting that the dermis is primarily a product of somite-derived cells. Similarly, myosepta must first be generated by the muscle cells themselves, because for most of development only the muscle cells are in contact with them. However, the non-muscle cells lining the myosepta in adults strongly express *ColA* (which muscle cells do not) and therefore likely function to strengthen and maintain the myosepta.

*ColA* is the sole clade A collagen in the amphioxus genome, and it is broadly expressed, including in the notochord and gill bars, two sites where cellular cartilage has been proposed to have originally evolved [36,46,47]. In vertebrates, collagen gene duplications are thought to be key events underlying the elaboration of connective tissues and origin of a mineralized skeleton. The clade A collagens are the most abundantly expressed type and include types I, II, III, and Va2. Collagen II expression is a hallmark of cartilage as well as notochord [48,49]. Dermis, tendons and ligaments are composed primarily of collagen type I, with lesser amounts of types III and V. Mineralized bone contains collagens I and V [45,50,51]. We find that amphioxus clade A collagen is expressed in all non-myotome somites and their derivatives throughout development and thus may have been similarly expressed in the last common ancestor with vertebrates. The diverse expression patterns of clade A collagen duplicates in vertebrate somitic compartments and their derivatives may represent sub-functionalization of an ancestral *ColA* expression pattern, *de novo* recruitment to vertebrate somitic compartments*,* or some combination of both. Other key changes, including regulation of collagen II by SoxE/Sox9 cassette (which occurred in stem vertebrates, and is common to agnathans and gnathostomes), have been described elsewhere [36,46,47] and recently [52].

### Potential homologies of vertebrate and invertebrate somitic compartments, and implications for the evolutionary origin of sclerotome

**Figure 18:**
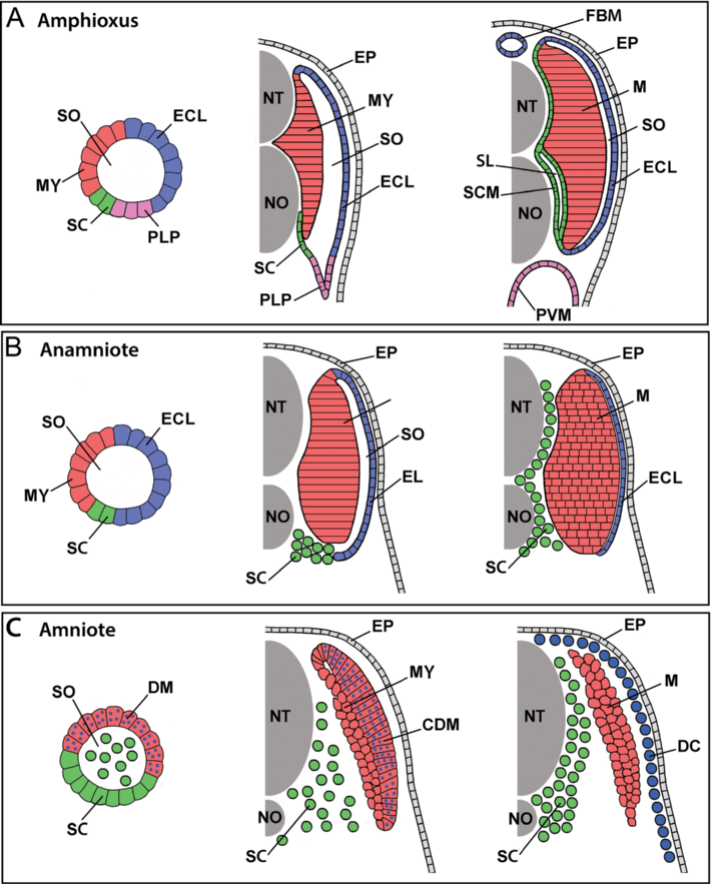
Comparison of somite development and somite compartment derivatives in **(A)** amphioxus, **(B)** an anamniote vertebrate, and **(C)** an amniote vertebrate. For each, somite organization is schematized at early (left panels), mid (middle panels), and late (right panels) developmental stages. See text for details. The schematics are shown unbent from their true chevron- or W-shape and for simplicity omit the ribs and ventral muscles (and in anamniotes, the myoseptal cells), which are derived from the somites and migrate ventrally into the lateral plate mesoderm. Other details are also omitted, including the difference between epaxial and hypaxial musculature and between phasic and tonic muscle fibers. Abbreviations: CDM, central dermomyotome; DC, dermal cells; DM, dermomyotome; ECL, external cell layer; EP, epidermis; FBM, fin box mesothelium; MY, myotome; NT, neural tube; NO, notochord; PLP, presumptive lateral plate; PVM, perivisceral mesothelium; SL, sclerocoel; SCM, scleromesothelium; SO, somitocoel; SC, sclerotome; M, trunk muscles. Major synonyms that others have used for the foregoing structures are listed in Table 1.

Figure 18 compares the organization and development of somites in amphioxus and vertebrates. In vertebrates, sclerotome arises on the ventral side of newly formed somites (Figure 18B,C). Despite differences across higher vertebrates (anamniotes *vs*. amniotes) in sclerotome position (proximity to the notochord), size, and induction mechanisms, there are many additional shared features. For example, vertebrate sclerotome is characterized by its ability to undergo EMT, its migration around midline structures and contribution to skeletal and connective tissue of the vertebrae and ribs (Figure 18B,C). It is also characterized by expression of *Pax-1/9* and *Twist-1*, and by containing cell populations expressing markers for cartilage (*Sox-9*) and for tendon and ligament (*Scx*) (reviewed in [1,2,4,53] and see [5,6]).

Since the skeleton is a vertebrate novelty, the sclerotome compartment may also have arisen in somites of early vertebrates. Alternatively, a sclerotome-like compartment may have pre-dated vertebrates but gained the ability to form skeletal tissue. Our observations of amphioxus non-myotome somite development reveal striking similarities with vertebrates, suggesting that most aspects of the sclerotome developmental program were in place before vertebrates evolved.

A comparison to teleost vertebral column development highlights the similarities in amphioxus. In teleosts (mainly studied in zebrafish, medaka, salmon, and trout) as in all vertebrates, the earliest axial support to develop is the notochord and its sheath. The notochordal sheath first appears as a basal lamina surrounding notochord cells, followed by a layer composed primarily of striated collagen II fibers and produced by the notochord itself [54-56]. After its formation, the collagenous layer of the sheath segmentally mineralizes to give rise to the chordacentra (unlike in amphioxus). Also unlike amphioxus, an outer elastin-rich layer, elastica externa, forms around the collagen layer. Teleost chordacentra are acellular and thus form differently from endochondral or dermal bone, and their formation is apparently independent of somite segmentation [54]. Subsequently, sclerotome-derived cells expressing collagen I migrate around the notochord and neural tube and give rise to the cellular skeletal tissue of the pericentrum and neural arches, respectively, which together comprise the vertebrae ([55] and references therein). The mode and timing of vertebral ossification varies by species and even AP region of the axial skeleton, and in some teleosts and more basal vertebrates (elasmobranchs), somite-derived cells also invade through the elastica externa and strengthen the chordacentrum, while in others, as in tetrapods, they remain outside the notochordal sheath [56-58]. Finally, it was recently shown that teleost myosepta initially form as acellular connective tissue between the myomeres and that they cellularize only late in development, via migration of mesenchymal cells from the sclerotome [5]. These myoseptal cells express collagen I (like the rest of the sclerotome and dermatome in teleosts), but they also express tendon markers including Scx and collagen Va2 [5,6].

Although less well studied, these processes appear conserved within the most basal vertebrates, agnathans, including formation of the notochordal sheath as well as the position and movement of sclerotome cells around midline structures, and expression of sclerotome (*Pax1/9*, *Twist*) and cartilage (*Sox9*) markers. Lamprey sclerotome also expresses *parascleraxis* (which duplicated in gnathostomes to *paraxis* and *scleraxis*), suggesting that it may also contain connective tissue progenitors [58-61]. This conservation of general sclerotome features from agnathans to teleosts (and in also in tetrapods, reviewed in [1]) suggests they are likely ancestral to vertebrates, despite many derived aspects of teleost development.

In amphioxus, as in fish, we find that the notochordal sheath first forms as a basal lamina, after which external layers of striated collagen arise, most likely derived from notochord. Later in amphioxus development, mesothelia derived from the sclerotome migrate around the midline structures and strongly express *ColA*; as we propose above, these may contribute to strengthening the axial extracellular connective tissue, similar to teleosts. Finally, the late appearance of collagen-expressing cells within amphioxus myosepta is also found in teleosts. These myoseptal cells’ earliest appearance near the medial and lateral ends of the myosepta, and early continuity with those somite-derived mesothelia, suggests a somitic origin.

Taken together, the position, migration path, likely fate as a connective tissue population that reinforces midline connective tissue, and potential contribution to the myosepta, are all features likely shared by the amphioxus ventral somite region and vertebrate sclerotome. We thus argue that these two structures are homologous, as Hatschek originally proposed. Furthermore, while detailed examination of expression domain and timing has yet to be performed, the amphioxus homolog of fish and tetrapod sclerotome marker, *twist*, is expressed in non-myotome somites, and *pax1/9* is expressed in somites as well [36,62].

These findings lead to a model for ancestral, invertebrate sclerotome that, like in amphioxus, arose in the ventral somite and contained connective tissue progenitors whose derivatives formed or reinforced the connective tissue support required for myomere-based movement. During the transition to vertebrates, this compartment would have evolved the ability to give rise to cartilage progenitors, while continuing to produce axial connective tissue as well. The first evolved vertebral elements were the neural and hemal arches, found in agnathans. In ancestral gnathostomes, sclerotome evolved to contribute to vertebral centra and ribs as well. While further modifications to sclerotome morphology and position can be seen during the course of vertebrate evolution, particularly in the transition to tetrapods, this dual fate, axial skeleton and axial connective tissue/tendon, is maintained.

Consistent with the idea of a cartilage progenitor fate evolving within a somitic connective tissue compartment, molecular genetic observations show that within the sclerotome of chick and mouse, axial tendon (*Scx* positive) and neural arch/rib cartilage (*Sox-9* positive) progenitors are co-mingled and overlap to some extent. Genetic lineage mapping reveals that descendants of Scx expressing cells contribute extensively to the neural arches and distal ribs and that tenocytes and chondrocytes near the intersection of tendon and cartilage share a common history of expressing both genes [63,64]. Further, axial tendon and neural arch/ rib cartilage fates are interconvertable when genetically manipulated, suggesting that antagonistic inductive mechanisms function to create distinct lineages from a common pool of cells [38,65]. In contrast, progenitors of axial tendon and centra cartilage do not appear to be interconvertable in the same way, and cells with a Scx expression history rarely contribute to centra, perhaps reflecting the different developmental and evolutionary histories of the neural arches/ribs and centra in tetrapods [64,65]. The mechanisms through which tendon and cartilage progenitor fates separate are not fully worked out, but it is known that both somitic tendon progenitors and neural arch/rib cartilage progenitors are induced by myotomally expressed Fgfs in amniotes and that somitic tendons also require Fgf signaling in teleosts [6,38,66,67].

## Conclusions

Here, we used a frequently sampled developmental series to characterize amphioxus somite and connective tissue development. We provide a revised model for amphioxus somite morphogenesis. Further, we propose that amphioxus non-myotome somite cells have a connective tissue identity and that their derivatives serve to strengthen the axial connective tissues which, in addition to the notochord, support myomere-based movement. Based on position, apparent migration path, and connective tissue identity, we propose that amphioxus non-myotome somites contain a cell population homologous to vertebrate sclerotome and that both evolved from an ancestral sclerotome containing connective tissue progenitors. It will be interesting to uncover the genetic changes necessary to derive a cartilage-producing cell population within a connective tissue somitic compartment.

## Abbreviations

adult: subadult
BS: budding somite
D: dermis
ECM: extracellular matrix
ECT: ectoderm
EL: external cell layer
EMT: epithelial-to-mesenchymal transition
END: endoderm
EP: epidermis
ES: epithelial somite
FBC: fin box coelom
GS: gill slit
HG: hindgut
M: muscle
MET: early metamorphosis stage larva
MS: myoseptum
MY: myotome
NEC: neurenteric canal
NEUR: neurula
NO: notochord
NP: neural plate
NS: notochordal sheath
NT: neural tube
post-met: post-metamorphic juvenile
PS: perineural sheath
PVC: perivisceral coelom
SL: sclerocoel
SO: somitocoel
TEM: transmission electron microscopy
